# Massive expansion of the calpain gene family in unicellular eukaryotes

**DOI:** 10.1186/1471-2148-12-193

**Published:** 2012-09-29

**Authors:** Sen Zhao, Zhe Liang, Viktor Demko, Robert Wilson, Wenche Johansen, Odd-Arne Olsen, Kamran Shalchian-Tabrizi

**Affiliations:** 1 Microbial Evolution Research Group (MERG), Department of Biology, University of Oslo, OSLO, N-0136, Norway; 2 Norwegian University of Life Sciences, Ås, N-1432, Norway; 3 Hedmark University College, Hamar, N-2306, Norway

**Keywords:** Calpain, CysPc domain, Unicellular eukaryote, Evolution, Gene family phylogeny, Protist

## Abstract

**Background:**

Calpains are Ca^2+^-dependent cysteine proteases that participate in a range of crucial cellular processes. Dysfunction of these enzymes may cause, for instance, life-threatening diseases in humans, the loss of sex determination in nematodes and embryo lethality in plants. Although the calpain family is well characterized in animal and plant model organisms, there is a great lack of knowledge about these genes in unicellular eukaryote species (i.e. protists). Here, we study the distribution and evolution of calpain genes in a wide range of eukaryote genomes from major branches in the tree of life.

**Results:**

Our investigations reveal 24 types of protein domains that are combined with the calpain-specific catalytic domain CysPc. In total we identify 41 different calpain domain architectures, 28 of these domain combinations have not been previously described. Based on our phylogenetic inferences, we propose that at least four calpain variants were established in the early evolution of eukaryotes, most likely before the radiation of all the major supergroups of eukaryotes. Many domains associated with eukaryotic calpain genes can be found among eubacteria or archaebacteria but never in combination with the CysPc domain.

**Conclusions:**

The analyses presented here show that ancient modules present in prokaryotes, and a few *de novo* eukaryote domains, have been assembled into many novel domain combinations along the evolutionary history of eukaryotes. Some of the new calpain genes show a narrow distribution in a few branches in the tree of life, likely representing lineage-specific innovations. Hence, the functionally important classical calpain genes found among humans and vertebrates make up only a tiny fraction of the calpain family. In fact, a massive expansion of the calpain family occurred by domain shuffling among unicellular eukaryotes and contributed to a wealth of functionally different genes.

## Background

Calpains are Ca^2+^-dependent cysteine proteases that regulate a multitude of intracellular processes by limited proteolysis of various substrates [1-3]. Defects in calpain function are associated with embryonic lethality in mice, muscular dystrophies in humans, gastropathy, tumorigenesis and neurogenesis disorders [4-7]. Furthermore, calpains function during embryonic development in *Drosophila* and mediate an environmental adapation to pH-dependent changes in fungi [8,9]. Plants contain only one calpain gene, Dek1, present in species ranging from the moss *Physcomitrella patens* to angiosperms, where it is essential for embryo development, and is proposed to have played a critical role in the evolution of differentiated multicellular plants [10-12].

The calpain family belongs to protease clan CA, and is classified into classical and non-classical forms based on domain architecture [3]. The classical calpains consist of four conserved domains: an N-terminal anchor helix (Nter), a catalytic protease core domain (CysPc) with the two subdomains PC1 and PC2, a C2-like domain (C2L) and a penta-EF-hand domain (PEF), designated here as Nter-CysPc-C2L-PEF. Non-classical calpains lack both the Nter and the PEF domain and may contain additional domains in combination with CysPc [2,3]. The majority of calpain research has focused on the three ubiquitously expressed classical calpains CAPN1 and CAPN2 in mammals and CAPN11 in birds. Both CAPN1 and 2 are closely related 80-kDa proteins and are highly conserved at the sequence and structural levels [13]. They can form separate heterodimers by binding to a common 30-kDa small regulatory subunit CAPNS1. Heterodimeric CAPNS1/CAPN1 and CAPNS1/CAPN2 have been named μ- and m-calpain, respectively, owing to the micromolar versus millimolar levels of Ca^2+^ required for their activation [1]. *In vivo*, calpain activity depends on the presence of three key catalytic amino acid residues (Cys, His and Asn) in the CysPc domain [14,15], although enzymes with substitutions in these residues do not always display loss of function [16]. Comparative analyses of representative animal genomes have revealed a relatively recent expansion of the calpain family and functional divergence among different paralogs [13,14,17,18]. Due to the modular nature of calpains, with many group-specific domains, phylogenetic tree construction based on the CysPc domain has proven to be the most efficient approach to understand the evolutionary divergence of classical and non-classical calpains [18,19]. For instance, the acquisition of the C-terminal PEF domain was shown, by this approach, to be a relatively recent event in the evolutionary history of classical calpains [20,21]. Using the evolutionary conservation of distinct calpain modules in diverse groups of eukaryotes, non-classical calpains were proposed to consist of several subfamilies, including the PalB subfamily found in humans, yeasts, fungi, insects and nematodes, the SOL subfamily for animals as well as the Dek1 subfamily that is represented in land plants [3]. The phylogenetic relationship among the members of non-classical calpains and the sequential evolution of calpain modules, however, remains unresolved.

Until now, studies on the diversity and evolutionary history of the calpain gene family have been focused on multicellular eukaryotes, including animals, land plants and fungi. Although calpain-like genes were also reported in several protozoan genomes such as those of the apicomplexan and kinetoplastid parasites [22,23], the great differences in gene number among those lineages (i.e. from one calpain-like gene in *Plasmodium falciparum* to 14 genes in *Trypanosoma brucei*) indicate a large variation of calpain diversity in single-celled eukaryotes. In fact, many unknown calpain genes may exist among hitherto unexplored unicellular organisms, some of which diverged early after eukaryotes arose and that display ancient morphological features and cellular structures [24,25]. Currently, almost all unicellular eukaryotes, animals and land plants have been assembled into six major groups (i.e. supergroups) on the basis of multi-gene phylogenies and deductions from cellular structures and chemistry [26-28]. These supergroups comprise Opisthokonta (i.e. animals, fungi and Choanozoa), Amoebozoa (e.g. pathogenic amoeboid *Entamoeba* and slime molds *Dictyostelium*), Excavata (e.g. amoeboflagellate *Naegleria*, parabasalid parasite *Trichomonas*, kinetoplastida parasites *Trypanosoma* and *Leishmania*), Plantae (e.g. green algae, red algae and land plants), SAR (i.e. Stramenopila (e.g. brown algae, diatoms and oomycota), Alveolata (e.g. apicomplexa, ciliates and dinoflagellates), and Rhizaria (e.g. cercozoa, foraminifera and radiolaria)) and a loose assemblage of Hacrobia (e.g. Haptophyta, Cryptophyta and Telonemia). Despite several attempts, the evolutionary relationships between these supergroups have not been completely resolved, but recent reports suggest that Plantae, SAR and Hacrobia are constituting a mega group [29], while Opisthokonta and Amoebozoa seem to form another mega group [30,31]. In addition to these established supergroups, there are a few orphan eukaryote lineages, such as *Thecamonas* and *Collodictyon*, that may occupy distinct positions in the tree of life [32,33]. They diverged very early in the history of eukaryotes and are crucial for understanding the evolution of eukaryotes. As a consequence, a comprehensive investigation of calpains distributed in these supergroups and deep diverged lineages would represent a key step towards a broader classificaton system as well as revealing the evolutionary events that contributed to the variation in this gene family. Importantly, reconstructing calpain phylogenetic profiles among these unicellular lineages may help illuminate the origin and evolution of proteolytic systems in eukaryotes and establish a practical framework on which experimental evidence can be compared between species.

In this study, we have searched for calpain genes in genomes representing a broad taxonomic sampling from all eukaryote supergroups with emphasis on unicellular organisms. We present a massively expanded calpain gene family, in which a large number of the new calpain genes are composed of many other domains than previously reported. Evolutionary inferences suggest that most of the calpain variants arose from the combinations of ancient domains through domain shuffling mechanisms. Domains derived more recently have contributed to the innovation of the calpain family by multiple independent insertion events. The majority of the new calpain variants are considered as non-classical types, implying that the classical calpains typical for animal and human genomes comprise only a small subset of the total gene family. The vast diversity of calpains described here provides a new framework for addressing the function of calpain genes in unicellular eukaryotes, and for elucidating the various levels by which these important proteins are regulated to prevent diseases or developmental defects in higher eukaryotes.

## Results and discussion

### A large diversity of domains and genes revealed in the calpain family

**Figure 1:**
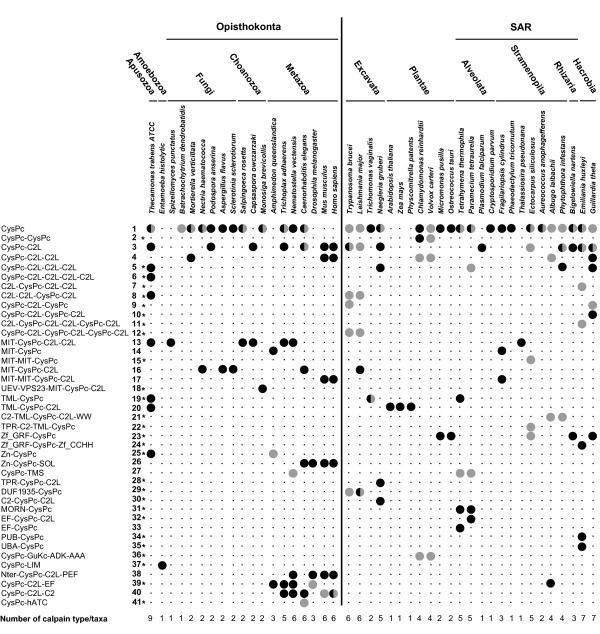
**Taxonomic distribution of calpain variants (left side) revealed by comparison of genomes from 44 eukaryotic species (top section).** Black circles indicate calpains with all three active site residues (Cys, His, Asn) intact in the CysPc domain. Grey circles indicate that the three CysPc active site residues are partially or completely substituted or missing. Half black and half grey circles indicate that some of detected calpains had all three residues, whereas others lacked at least one of the active residues. Black dots show that the calpain domain combination is absent. * indicates a novel domain combination identified in this work. All calpains are listed with accession numbers in Additional file 1: Table S1.

Our survey of calpain diversity identified a total of 41 different domain arrangements, of which 28 have not been previously reported (Figure 1). Combinations between CysPc and 24 other domains such as hATC (hAT family of dimerization domains), LIM (Zinc-binding domains present in Lin-11, Isl-1 and Mec-3), TPR (Tetratricopeptide repeats), WW (a domain with two highly conserved tryptophans) and Zf_GRF (a GRF zinc finger) reveal this huge variation in calpain members (see Table 1). By searching protein family databases, we found homologs of 16 of these domains in either eubacteria or archeabacteria (Table 1), implying the majority of domains are ancient and have participated in the formation of other genes than calpains before the origin of eukaryotes. Innovation in the calpain family has therefore taken place by adding both ancient and novel domains to the N- or C- terminus of CysPc with variable types, numbers and orders (Figure 1 and Additional file 1: Table S1).

**Table 1:** The distribution of domains identified in calpain genes in the three domains of life

**Abbreviation**	**Description**	**Eukaryotes**	**Eubacteria**	**Archaebacteria**	**Function**	**Reference**
ADK	Adenylate kinase	*	*	*	Cellular energy homeostasis	Berry MB et al., 1998
C2	Protein kinase C conserved region 2 (CalB)	*	*	*	Signal transduction or membrane trafficking	Ponting CP et al., 1996
C2L	Calpain domain III	*			Regulation of calpain activity	Sorimachi H et al., 2011
CysPc	Calpain-like protease catalytic domain	*	*		Apoptosis, membrane fusion, cell motility, and signal transduction	Sorimachi H et al., 2011
DUF1935	Domain of unknown function 1935	*			Unknown	Bateman A et al., 2010
EF	EF hands	*	*		Ca^2+^-binding	Nakayama S et al., 1992
GuKc	Guanylate kinase homologs	*	*	*	Active Guanylate kinase catalyze ATP-dependent phosphorylation of GMP to GDP	Kuhlendahl S et al., 1998
hATC	hAT family dimerization domain	*	*		Proposed to participate in regulation of transcription	Rubin et al., 2001
LIM	Zinc-binding domain present in Lin-11, Isl-1, Mec-3	*	*	*	Zn^2+^-binding, mediate protein-protein interactions	Perez-Alvarado GC et al., 1992
Nter	N-terminal anchor helix	*			Participate in classical calpain conformation change	Sorimachi H et al., 2011
MORN	Membrane Occupation and Recognition Nexus	*	*		Unknown	Takeshima et al., 2000
PEF	Calpain domain IV	*			Ca^2+^-binding	Sorimachi H et al., 2011
PUB	PUB domain	*	*		AAA ATPase binding domain	Allen MD et al., 2006
SOL	Small optic lobes	*			Unknown	Kamei M et al., 1998
TM(L/S)	(Long or Short) Transmembrane motifs	*	*		Unknown	Corall and Ersfeld K 2007
TPR	Tetratricopeptide repeats	*	*	*	Mediate protein-protein interactions	Palmer CP et al., 2004
UBA	UBA/TS-N domain	*	*	*	Ubiquitin binding	Dieckmann T et al., 1998
UEV	UEV domain	*			Functions in both HIV-1 budding and the vacuolar protein sorting (VPS) pathway in human	Wagner KU et al., 2003
Vps23	Vps23 core domain	*			Stable ESCRT-I complex	Teo H et al., 2006
WW	Domain with two highly conserved tryptophans	*			Binds proline-rich polypeptides	Bork et al., 1994
Zf_GRF	GRF zinc finger	*	*		Proposed in nucleic acid binding	Krishna SS et al., 2001
Zf_CCHH	Zinc-finger (CX5CX6HX5H) motif	*			DNA strand break repair, DNA metabolism	Iles N et al., 2007
Zn	Zinc finger domain in Ran-binding proteins	*	*	*	RanGDP binding	Krishna SS et al., 2001
AAA	AAA ATPase domain	*	*		ATPases associated with diverse cellular activities	Hanson PI et al. 2005
MIT	Microtubule interacting domain	*		*	Intracellular trafficking	Phillips SA et al., 2001

Star * indicates the type of domain is present in Eukaryotes, Eubacteria or Archaebacteria.

Among the investigated species, *Thecamonas trahens,* a lineage that phylogenetically belongs to Apusozoa, shows the highest number of different calpain paralogs. It has 12 genes that encode nine calpain variants (Figure 1 and Additional file 1: Table S1). Three other species, the single-celled phytoplankton *Emiliania huxleyi,* (Hacrobia) the amoeboa-flagella *Naegleria gruberi* (Excavata)and the ciliate *Paramecium tetraurelia* (SAR) also contain a large number of genes encoding seven, five and four calpain variants, respectively. In contrast, one single calpain was found in all land plants (named Dek1), *Entamoeba histolytica*, *Spizellomyces punctatus, Plasmodium falciparum*, *Cryptosporidium parvum* and *Thalassiosira pseudonana*. No CysPc domain was detected in *Giardia intestinalis*, *Dictyostelium discoideum, Chlorella NC64A* or red algae (*Cyanidioschyzon merolae*), even if we loosened the threshold (e-value < 0.01) in the BLAST searches. Thus, we found a large variation in calpain gene numbers in the 44 representative taxa. Even closed related species, such as species in genera *Trichomonas* and *Giardia*, have very different calpain gene content.

### Four ancient eukaryotic calpain domain architectures

**Figure 2:**
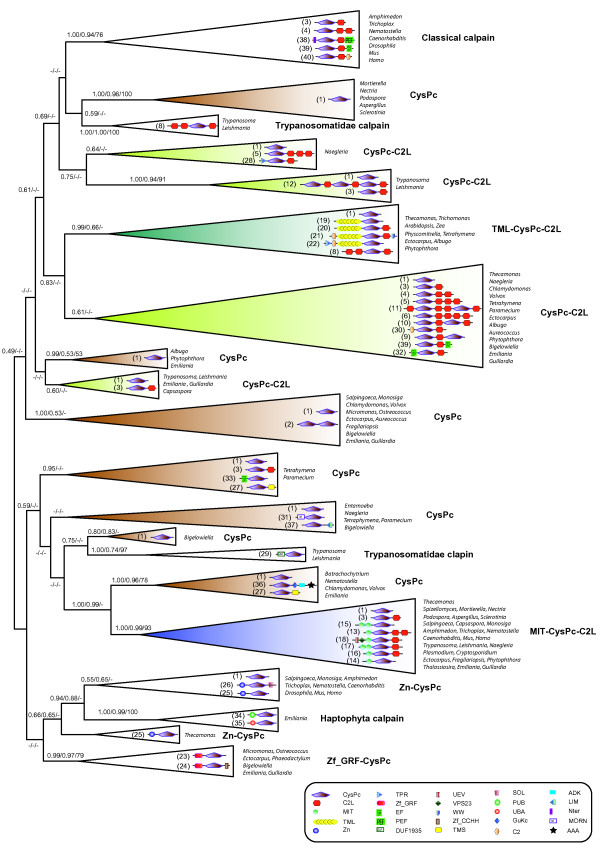
**Eukaryotic calpain phylogeny reconstructed by Bayesian inference of the CysPc domain sequence.** Several lineages that share similar domain combinations are collapsed into major clades for details of species names and protein domains, see Figure 1 and Additional file 2: Figure S1. Typical domain organization is schematically displayed in each clade. The names of domains are shown on the bottom with different symbols. Only the genus names of sampled taxa are listed. The clades representing four proposed ancestral domain architectures are color-coded (i.e. CysPc in dark brown, CysPc-C2L in blue, MIT-CysPc-C2L in light green and TML-CysPc-C2L in dark green). For each node, statistical support values are marked (numbers from left to right: Bayesian posterior probabilities (PP) inferred under LG /CAT models and maximum-likelihood bootstraps (% BP) inferred using PROTGAMMALG model). Dashes ‘-’ indicate support values < 50% BP or 0.5 PP.

**Figure 3:**
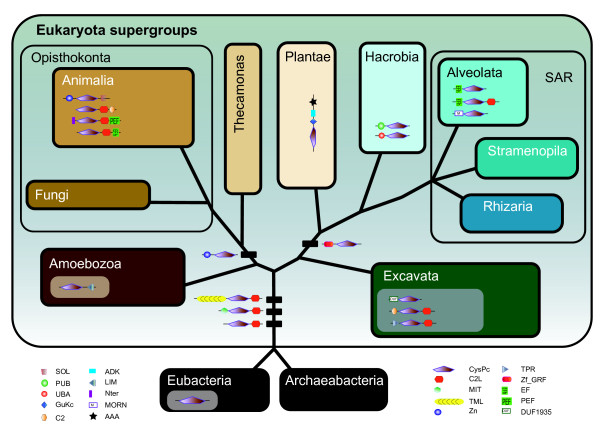
**Proposed origin of calpain domain architectures mapped on the global eukaryote phylogeny [**[26,28][33]**].** Black bars on the branches indicate the hypothetical origin of specified domain combinations. Calpain variants found in one taxon or among closely related species are schematically displayed within the supergroup rectangles, which likely constitute lineage-specific domain combinations.

Of all 41 calpain types, we identified that 13 variants are present in more than one supergroup. For instance, types 1 (CysPc), 3 (CysPc-C2L) and 4 (CysPc-C2L-C2L) show a scattered taxonomic distribution (Figure 1). In order to determine whether these calpain genes evolved only once or on multiple occasions, we reconstructed their evolutionary relationships based on the alignment of CysPc. Based on the distribution of domain combinations across the eukaryote tree and the similarity of domain components in various genes, we propose that four calpain architectures CysPc, CysPc-C2L, MIT-CysPc-C2L and TML-CysPc-C2L originated early in the evolutionary history of eukaryotes Figure 2, Figure 3 and Additional file 2: Figure S1.

First, the type of calpain containing only CysPc is likely the most ancient form, both because it has the broadest distribution across eukaryote supergroups, and is the only form of calpain present in eubacteria [18]. As seen from Figure 1, this variant is found in Opisthokonta, Excavata, SAR, Plantae, Hacrobia and *Thecamonas*. The clustering of CysPc calpain into seven different clades (Figure 2 and Additional file 2: Figure S1), suggests that several ancient paralogs were established by multiple independent duplications and subsequently diverged into distinct forms. Interestingly, within some of the CysPc clades, a few sequences are composed of multiple domains (e.g. types 36 and 27), implying that the different CysPc paralogs have recruited other domains along the evolution of the eukaryotes at several independent occasions. In order to investigate whether the eukaryotic CysPc paralogs derived from bacteria once or many times, we added several prokaryote CysPc domains to the alignment and repeated the phylogenetic reconstruction (see Additional file 3: Figure S2). In the resulting tree, the bacterial paralogs are weakly recovered as a monophyletic group (BP <50%, PP_LG = 0.68 and PP_CAT = 0.53 in Additional file 3: Figure S2; BP is bootstrap support; PP_LG/PP_CAT is the bayesian posterior probability inferred under LG/CAT model) with affinity to one of the eukaryotic CysPc clades, indicating that all eukaryote CysPc variants may have evolved from a single bacterial paralog.

The calpain type 3 (CysPc-C2L) probably arose soon after the early duplication of domain CysPc based on its presence in nearly all supergroups (e.g. Opisthokonta, Excavata, Hacrobia and SAR). In addition, it was found in *Thecamonas*. This gene may have been lost early in Amoebozoa and Plantae (Figure 1 and Figure 2). Several CysPc-C2L calpains show variable numbers of C2L (i.e. types 4–6), suggesting multiple tandem duplications of C2L. Since Figure 2 and Additional file 2: Figure S1 both show CysPc-C2L calpains as a paraphyletic clade, independent C2L domains may have been added to the different CysPc paralogs early in eukaryote evolution. Furthermore, the phylogeny indicates that CysPc-C2L calpains have subsequently recruited other types of domains on several independent occasions giving rise to new calpain genes (Figure 2 and Additional file 2: Figure S1).

The third ancestral calpain subfamily is composed of the MIT-CysPc-C2L variants (i.e. type 13–18; Figure 1). The members in this subfamily are present in most eukaryote supergroups, and they are strongly recovered as a monophyletic clade in the phylogeny (93% BP, 0.99 PP_LG and 1.00 PP_CAT in Figure 2 and Additional file 2: Figure S1). The MIT-CysPc-C2L calpains may have been formed by a integration of a microtubule interacting and transport (MIT) module at the N-terminus of the CysPc-C2L. Our survey revealed a broad distribution of this architecture in Opisthokonta and Apusozoa (Figure 1). In addition, we identified several calpains with highly divergent MIT domains in the genomes of *Ectocarpus, Thalassiosira, Fragilariopsis* and *Leishmania* (Additional file 2: Figure S1). This broad distribution of the MIT domain among eukaryotes indicates that it might have been present since the very early stages of calpain evolution. In unicellular Opisthokonta and deeply diverging animals, a few taxa (i.e. *Thecamonas*, *Capsaspora* and *Nematostella*) have one duplicated C2L at the C-terminus, forming MIT-CysPc-C2L-C2L. In contrast, the presence of one duplicated MIT domain at the N-terminus of CysPc is observed in vertebrates (BP = 86%, PP_LG = 0.99 and PP_CAT = 0.81; Additional file 4: Figure S3), implying that the calpain architecture in the ancestral vertebrate may have been MIT-MIT-CysPc-C2L/C2L.

The fourth most ancient architecture is represented in calpain types 19–22, consisting of a large transmembrane domain TML, with more than 15 transmembrane segments linked to the N-terminus of CysPc-C2L. These are found in the Excavata, Plantae and SAR supergroups as well as in *Thecamonas*, and are inferred to be monophyletic in the calpain phylogeny (BP < 50%, PP_LG = 0.99 and PP_CAT = 0.66; Figure 2 and Additional file 2: Figure S1). Recent multi-gene phylogenomic trees robustly support *Thecamonas* branching as a sister to Opisthokonta [31]. Thus, the broad presence of this particular calpain type implies that the TML-containing calpain gene most likely originated very early but was subsequently lost in the common ancestors of Amoebozoa and Opisthokonta (Figure 1 and Figure 2). Interestingly, gene expansions within this subfamily are observed in the Excavata species *Trichomonas*. Seven paralogs of TML calpains in *Trichomonas* are robustly recovered as a monophyletic group. They all show the absence of the C2L domain, indicating that it may have been lost in a single event before the multiple duplications of the gene (Additional file 2: Figure S1). In contrast to other lineages, only one type of calpain gene with a TML domain (TML-CysPc-C2L), named Dek1, is found in land plants ranging from *Physcomitrella* to *Oryza*. We did not identify any Dek1-like calpains carrying a TML module in the genomes of *Chlamydomonas, Volvox, Micromonas* or *Ostreococcus.* Altogether, the wide distribution of the TML domain among eukaryotic calpains suggests that this domain combination was likely formed before the split of land plants and green algae, then secondarily lost in the early evolution of the Chlorophyta green algae.

### Implications of an alternative eukaryote root

The evolutionary events that contributed to the assembly of these four ancient calpain subfamilies are suggested to have occured before the bipartition of ‘Opisthokonta-Amoebozoa’ and ‘Excavata-Plantea-SAR-Hacrobia’ (Figure 3) in accordance with the previously proposed hypothesis for the eukaryotic root [28,34]. However, we still deduced these four calpain types as ancient forms if we change the root of eukaryotes to the Excavata as recently proposed [35] (See Additional file 5: Figure S4), because of the broad taxonomic distribution of the four subfamilies and their presence in key Excavata species.

### Lineage specific gene innovations

Several calpain domain architectures appear to be specific for one or more eukaryote supergroups, and are therefore most likely generated before the massive speciation in these lineages along the eukaryote tree. One of these, Zn-CysPc, seems to be possessed by Opisthokonta (Additional file 3: Figure S2 and Figure 3; BP < 50%, PP_LG = 0.55 and PP_CAT = 0.65) while another domain combination, Zf_GRF-CysPc, is shared by SAR, Hacrobia and Plantae (BP = 79%, PP_LG = 0.99 and PP_CAT = 0.97; Additional file 3: Figure S2 and Figure 3). Both the Zn and Zf_GRF domains have multiple finger-like protrusions that make tandem contacts with their target molecules, but their distinct motifs and distributions among eukaryotes suggest independent origins (Figure 3).

Novel domain architectures are also found to be specific for certain groups of eukaryotes (Figure 3). For instance, an insertion of one SOL module to the C-terminus of Zn-CysPc was observed only in *Caenorhabditis*, *Drosophilia*, *Homo* and *Mus*, indicating that Zn-CysPc-SOL may have formed after the origin of Metazoa. Moreover, our survey shows no indications of the classical calpain architecture (Nter-CysPc-C2L-PEF) outside the Metazoa and hence strengthens the notion that these genes are animal-specific [3] (Additional file 6: Figure S5). Both Nter and PEF were likely added to the N- and C-termini, respectively, of CysPc-C2L variants in the last common ancestor of Metazoa [2]. In some calssical calpains, PEF domains contribute to protein dimerization (either homodimerization or heterodimerization). However, based on recent data, it seems that the occurrence of PEF and Nter domains is not directly associated with dimerization process in all classical calpains. We also found that two calpain variants with an EF-hand module are present in *Paramecium* (type 32: EF-CysPc-C2L) and *Tetrahymena* (type 33: EF-CysPc), respectively (Figure 1). However, their motifs are not identical to PEF, indicating independent insertion of the EF-hand domains. The EF-hand modules found in ciliate calpains may be involved in binding calcium ions [36], but it is uncertain what concentration of calcium ions is required for regulating these calpains.

A few calpain domain combinations seem to have been derived very recently in a smaller group of organisms. For instance, only the closely related species *Trypanosoma* and *Leishmania* share the domain architecture DUF1935-CysPc; hence this combination is exclusive for Trypanosomatidae (BP = 97%; PP_LG = 1.00 and PP_CAT = 0.74; Figure 2 and Figure 3). Calpain type 36, composed of CysPc-GuKc-ADK-AAA, is only identified in *Chlamydomonas* and *Volvox.* It may have been created by the insertion of domains GuKc, ADK and AAA into the C-terminus of the CysPc paralog, therefore representing a gene unique for Chlorophyta green algae (Figure 3). The gain of PUB and UBA modules at the N-termini of *Emilinia* CysPc paralogs shows that the two ombinations are probably unique to this particular group of Haptophyta. The close relationship of PUB-CysPc and UBA-CysPc in the phylogenetic tree (Figure 3) implies they probably share a common origin (BP = 100%, PP_LG = 1.00 and PP_CAT = 0.99; Additional file 2: Figure S1). Two other calpain variants, one with simple transmembrane motifs (TMS) and the other with MORN, are both found in *Tetrahymena* and *Paramecium*. They seem to be shared by ciliates (BP = 88%, PP_LG = 1.00 and PP_CAT = 0.88 for CysPc-TMS; BP = 94%, PP_LG = 1.00 and PP_CAT = 1.00 for MORN-CysPc; Additional file 2: Figure S1). As we show above, many other domains were added to the N- or C- termini of CysPc paralogs by domain-wise evolutionary events and formed lineage specific arrangements [37-39].

### Two patterns of domain shuffling in the calpain gene family

Altogether, the data reveal two different patterns in the evolution of the calpain gene family. First, the majority of domains in calpains are ancient and have existed as part of other genes and not in combination with CysPc since before the origin of eukaryotes. The early evolution of the calpain gene family therefore most likely occurred by domain shuffling of ancient domains. During the evolutionary history of eukaryotes, domains have been added successively to create increasingly complex genes. In addition, there are several examples of secondary losses or modification of domains. For instance, in the MIT-calpain clade in Figure 2 and Additional file 2: Figure S1, several sequences either lack or have highly divergent MIT domains, indicating that the MIT domains have been secondarily lost or modified at several occasions. Second, in contrast to this dominating pattern of evolution, we also find nine domains in the calpains which are not present among prokaryotes (see Table 1). Such domains have contributed to the generation of genes unique for specific supergroups of eukaryotes and hence constitute lineage-specific paralogs. Genes specific to certain eukaryote groups therefore seem to have evolved either by combining only ancient domains, such as UBA, GuKc and ADK, or by combining a mix of ancient and novel domains, such as the Trypanosomatidae-specific DUF1935.

### Uncover new functions in non-classical calpains

Up to now, insight into the regulatory mechansims and physiological functions of calpains has mainly been based on studies of the classical calpains in mammals. Obviously, the discovery of a large variety of non-classical calpains creates expectations of many new functions and regulatory mechansims for calpains yet to be uncovered. One important source for new functions lies in the large number of non-classical calpains where one or more of the three amino acid residues essential for enzyme catalytic function have been replaced (grey and half grey circles in Figure 1 and more details in Additional file 1: Table S1). In particular, the occurrence of such substitutions is prominent for *Emilinia, Ectocarpus* as well as Trypanosomatidae [22], where more than 50% of the calpains variants show partial or complete loss of catalytic site residues. The findings support earlier suggestions that these variants may have divergent functions that do not rely on proteolytic processing [2]. This is demonstrated by the recent finding that links CAPN6 to a non-proteolytic function in eutherians, where the active site Cys is replaced with Lys. The non-classical CAPN6 functions as a microtubule-stabilizing protein [40]. Moreover, CAPN5, CAPN6, CAPN7 and CAPN10 have been suggested to share a similar function in the regulation of microtubule stability due to their comparable architectures with tandem C2L/C2 domains at the C-terminus of CysPc [41]. Thus, they are classified together in the PalB subfamily. Yet, this grouping is not supported by the present analysis. As discussed above, CAPN7 (MIT-MIT-CysPc-C2L-C2L) shows an ancestral origin (Figure 2 and Additional file 2: Figure S1). In contrast, CAPN6 and CAPN10 seem to have arisen more recently. CAPN6 is placed as sister to CAPN5, supported by 64% BP, 0.99 PP_LG and 0.97 PP_CAT (Additional file 6: Figure S5). Both of them are grouped with CAPN10 close to the clade of the classic calpains (CAPN13 and CAPN14; Additional file 6: Figure S5). Therefore, despite the common domain features, the clustering of CAPN7, CAPN6 and CAPN10 in different groups, indicate that they may have acquired different functions.

The non-classical calpain Dek1 is localized to the plasma membrane where it is proposed to be activated by a transmembrane anchor [12]. It has been suggested that the Dek1 homolog of the protist *Tetrahymena* may have been acquired by lateral gene transfer from a green alga-type endosymbiont of ciliates [2]. With the addition of more TML-calpains to the phylogeny, the Dek1-like calpain in *Tetrahymena* now clusters weakly as a sister to those in *Thecamonas* (Additional file 2: Figure S1). This result does not support the lateral gene transfer hypothesis to explain the expansion of Dek1-like genes in taxa outside land plants. On the contrary, our phylogeny indicates an early origin of Dek1-like genes and subsequent divergent evolution of the amino acid sequences. Interestingly, one mammalian non-classical calpain (CAPN15) shows significant similarity to the CysPc domain of Dek1-like calpains (almost 40% amino acid sequence identity) [18]. However, the phylogenetic tree does not indicate that they share the same origin. In addition, we observed complicated multiple domain architectures of TML-calpains in the taxa of Stramenopila. They share a common arrangement C2-TML-CysPc. For *Ectocarpus*, the TPR domain was inserted to the N-terminus of C2-TML-CysPc. By contrast, in *Albugo* and *Phytophthora*, the WW domain was anchored to the C-terminus of the sequence. Overall, we conclude from these studies that a high degree of sequence divergence and a variety of multiple domain architectures in TML-calpains provide a promising system to elucidate the functional significance of their membrane association.

The simplest calpain variant consisting only of the single cysteine protease domain CysPc is rare in multicellular organisms. Rather, it is prevalent in primitive protists and algae. Likewise, we observed that a few non-classical calpains do not contain the C2L domain, while all of non-classical calpains lack the PEF domain. The regulation of these calpain forms is currently unknown. However, based on the observation that the two Ca^2+^ binding sites of CysPc are implicated in the regulation of classical calpains, one possibility is that Ca^2+^ is the main regulator of these enzymes as well [42,43]. Classical calpains, on the other hand, have evolved multiple levels of control over their proteolytic activities imposed by interactions between CysPc and C2L, PEF, the small regulatory subunit as well as the calpastatin inhibitor [44]. Based on the elucidation of the evolutionary history of calpains presented here, it is now possible to study each control step separately in the different calpain variants shown in Figure 1. Ultimately, this insight may contribute to novel strategies for controlling calpains in human pathologies and further the progress of research into calpain function in general.

## Conclusion

Calpains in vertebrates and land plants are known to be crucial for a multitude of physiological and intracellular processes. Here, we report a massive expansion of the calpain gene family in unicellular eukaryotes, many of which arose by combining CysPc with protein domains previously unrecognized in this family. Phylogenetic inferences support the hypothesis that four calpain gene variants may have been formed in the early evolution of eukaryotes by assembly of ancient domains that were already present among prokaryotes. The lineage-specific calpain genes, however, were formed through shuffling of both ancient and novel eukaryote-specific domains. Overall, comparative genomic analyses of this family establish a framework for understanding the evolutionary mechanisms involved in the origin and expansion of eukaryote calpain genes, and it provides a basis for investigating cellular functions of calpain genes.

## Methods

### Taxonomic sampling

We sampled 34 unicellular eukaryotic organisms for surveying and characterizing calpain diversity. These taxa represented a wide selection from the proposed supergroups including the Choanozoa (i.e. *Capsospora owczarzaki* and *Salpingorca rostta*), the basal fungi (i.e. *Spizllomyces punctatus*, *Batrachochytrium dendrobatidis* and *Mortierella verticillata*), the pathological parasites (i.e. kinetoplastid, apicomplexan, *Trichomonas vaginalis* and *Entamoeba histolytic*), green algae (i.e. *Chlamydomonas reinhardtii* and *Volvox carteri*), brown algae (i.e. *Ectocarpus siliculosus*, *Phytophthora infestans* and *Thalassiosira pseudonana*), phagotrophic protists (i.e. *Thecomonas trahens*, *Tetrahymena thermophila* and *Paramecium tetrautelia*) and marine phytoplankton (i.e. *Bigelowiella nartens*, *Guillardia theta* and *Emiliania huxleyi*). In addition, three early diverged multicellular species (i.e. *Amphimedon queenslandica*, *Trichoplax adhaere*ns and *Nematostella vectonsis*) in Metazoa together with seven representative land animals and plants were involved in taxonomic sampling as well. For a full overview of the species in our analyses and how they are related to the corresponding supergroup, see Additional file 1: Table S1.

### Comparative genome analyses

A primary BlastP or tBlastN [45] search was performed using the CysPc domain of CAPN1 and CAPN2 as query sequences against protein and genome databases of the 44 aforementioned eukaryotic organisms (Genbank eukaryote genomes, non-redundant databases, the Institute for Genomic Research, Joint Genome Institute Resource, the Broad Institute of Harvard and MIT) to identify genes containing the CysPc domain (e-value < e^-10^). We used a relatively strict criterion to collect calpain-like genes with a high-quality sequence. Specifically, we included sequences that showed more than 40% overlap with the CysPc query. Here, one gene encoding a ‘calpain-like cysteine protease’ was found in *Dictyostelium discoideum* AX4 [46], but this sequence was excluded from our dataset due to lacking significant hits in the CysPc domain region. Each calpain-like sequence was then searched against the protein conserved domain database (CDD), SMART database and the Pfam database to annotate domain modules [47-49]. We only selected domain modules with a significant annotation in Pfam, SMART or CCD. But for MIT module detection, we loosened the threshold and included a few sequences with derived domains in order to better understand the origin of MIT calpains and the pattern of MIT loss and modifications. Since boundaries of a domain region annotated by the three database searches were not identical, we used the consensus of all significant domains predictions. In cases where domains of different characteristics overlapped, we regarded the annotation too uncertain to be included in the presentation. For the CysPc domain, we illustrated the domain based on the Pfam annotation only since the annotations from different databases showed a sequence overlap > 90%. For other domains, we used the consensus from the annotations of different databases to define the domain. When different annotation approaches identified variable number of domain repeats, we only show one copy to represent that domain. For the identification of the three catalytic sites in CysPc domain, we combined the prediction given by the Pfam database and the pairwise comparison of each protein studied here vs. the classical CysPc domain. Transmembrane motifs were predicted by the TMHMM Server v. 2.0 [50]. The detailed information (i.e. accession number, the type of domain combination and number of replacements in the three catalytic sites) of all calpain sequences presented here are listed in Additional file 1: Table S1.

### Construction of the alignment

The CysPc sequences were then aligned by MAFFT using the L-INS-i algorithm [51], following by manual editing in MacClade 4.0 [52]. Only positions that were unambiguously aligned were included in the further analyses. Alignments that sampled 259 sequences were initially analyzed, and then reduced by removing 12 sequences (for details, see Additional file 1: Table S1) because of their highly divergent sequences prone to generating phylogenetic artefacts. The final alignment consisted of 247 sequences and 202 sites available at http://www.mn.uio.no/bio/english/people/aca/kamran/data/Calpain_final_247_accession.dat (the original alignment before editing can be downloaded from http://www.mn.uio.no/bio/english/people/aca/kam-ran/data/Calpain_final_247_accession_original.dat). ProtTest 3.0 was used for amino acid substitution model selection using the Akaike Information Criterion (AIC) to choose the best-fitting tested model (LG + GAMMA) for phylogenetic analyses [53].

### Phylogenetic analyses

Reconstruction of maximum likelihood (ML) phylogenies from the calpain sequence alignment was performed using RAxML v7.2.6 [54]. For multiple CysPc domains found in calpain sequences (e.g. types 2, 9, 10 and 12 in Figure 1), all of them were included in the phylogenetic analyses. The best topology was determined after 100 heuristic searches starting from 100 different random trees under the PROTGAMMALG model. Statistical support was evaluated with 500 bootstrap pseudo-replicates under the same model as in the initial tree search. Bayesian phylogenies were inferred by Phylobayes v3.2 under both the LG model and the CAT mixture model in combination with four gamma categories for approximating the rate heterogeneity across sites [55]. Two independent Markov Chains Motor Carlo (MCMC) starting from two random trees were run for 50,000 cycles with one tree being sampled every cycle. Consensus topology and posterior probability values were calculated from saved trees after discarding 10,000 cycles as burn-in. Convergence between the two chains was ascertained by examing the difference in frequency for all their bipartitions (maxdiff < 0.15 in all analyses).

## Competing interests

The authors declare that they have no competing interests.

## Authors’ contributions

OAO and KST and conceived, designed and supervised this study. ZL and SZ collected data and performed analyses. SZ, ZL, RW, OAO and KST interrepted the data. SZ, ZL, KST, OAO, RW contributed to drafting the manuscript. VD and WJ read and revised the mansucript. All authors read and approved the final manuscript.

## Supplementary Material

Additional file 1 **Table S1.** Accession numbers, domain combination of calpains. Click here for file

Additional file 2 **Figure S1.** Eukaryotic calpain phylogeny inferred from the CysPc domain alignment (247 calpain sequences and 202 positions). The phylogeny is obtained from the consensus between two independent Bayesian inferences. For each node, support values are marked (numbers from left to right: Bayesian posterior probabilities (PP) inferred under LG /CAT models and maximum-likelihood bootstraps (% BP) inferred using PROTGAMMALG model) if all are more than 80% BP and 0.8 PP (filled circle) or more than 50% BP and 0.5 PP (open circle). Dashes ‘-’ show the support values are marked< 50% BP or 0.5 PP. For multiple CysPc domains found in the calpain sequence,1st, 2nd and 3rd indicate their orders from N-terminus to C-terminus of the calpain sequence. The domains marked by ‘*’ indicate their identities only have marginal significance of e-value in domain database research due to high sequence divergence. The branches and clades are assigned to the numbers that represent specified domain combinations shown in Figure 1. Click here for file

Additional file 3 **Figure S2.** Eukaryotic calpain phylogeny rooted by the bacterial outgroup (246 eukaryotic + 15 prokaryotic calpain sequences; 202 positions). The phylogeny is obtained from the consensus between two independent Bayesian inferences. For each node, support values are marked (numbers from left to right: Bayesian posterior probabilities (PP) inferred under LG (left) /CAT (middle) models and maximum-likelihood bootstraps (% BP) inferred using PROTGAMMALG (right) model) if all are more than 80% BP and 0.8 PP (filled circle) or more than 50% BP and 0.5 PP (open circle). Dashes ‘-’ show the support values < 50% BP or 0.5 PP. For multiple CysPc domains found in the calpain sequence,1st, 2nd and 3rd indicate their orders from N-terminus to C-terminus of the calpain sequence. The domains marked by ‘*’ indicate their identities only have marginal significance of e-value in domain database research due to high sequence divergence. The branches and clades are assigned to the numbers that represent specified domain combinations shown in Figure 1. The alignment used to build this phylogeny can be downloaded from http://www.mn.uio.no/bio/english/people/aca/kamran/data/Calpain_final_261_accession_outgroup.dat. Click here for file

Additional file 4 **Figure S3.** Phylogeny of the calpain genes with MIT modules. The tree is obtained from the consensus between two independent Bayesian inferences. Support values are marked at the nodes, and numbers from left to right represent Bayesian posterior probabilities (PP) inferred under CAT /LG models and the maximum-likelihood bootstraps (% BP) inferred using PROTGAMMALG model in RAxML v7.2.5. All support values more than 80% BP and 0.8 PP or more than 50% BP and 0.5 PP are shown as full or open circles. Dashes ‘-’ show the support values < 50% or 0.5 PP. The MIT calpain genes found in *Tetraodon nigroviridis, Takifugu rubripes, Gasterosteus aculeatus, Gadus morhua, Salmo salar, Danio rerio, Oryzia latipes, Gallus gallus, Dasypus novemcinctus, Ailuropoda melanoleuca, Sus scrofa, Canis familiaris, Bos taurus and Rattus norvegicus* are added to this analysis. The domains marked by ‘*’ indicate their identities only have marginal significance of e-value in domain database research due to high sequence divergence. The branches and clades are assigned to the numbers that correspond to specified domain combinations shown in Figure 1. The alignment used to reconstruct the phylogeny of MIT calpains can be downloaded from http://www.mn.uio.no/bio/english/people/aca/kamran/data/Calpain_MIT_accession.dat. Click here for file

Additional file 5 **Figure S4.** Proposed origin of calpain superfamily domain combinations shown on the global eukaryote phylogeny. The tree is rooted according to Cavalier-Smith T 2010. Biol Lett 6(3):342-345. Red bars on the branches indicate the hypothetical origin of specified domain combinations. Calpain variants only found in one taxon or among closely related species are marked in red within the supergroup rectangles, which likely constitute lineage-specific domain combinations. Click here for file

Additional file 6 **Figure S5.** Phylogeny of calpains in Metazoa. The topology is obtained from the consensus between two independent Bayesian inferences. The representative taxa sampled here include *Homo species, Mus musculus, Gallus gallus, Xenopus laevis, Danio rerio, Nematostella vectensis, Drosophila melanogaster, Caenorhabditis elegans, Trichoplas adhaerens and Amphimedon queenslandica*. Support values are marked at the nodes, and number from left to right represent Bayesian posterior probabilities (PP) inferred under CAT /LG models and the maximum-likelihood bootstraps (% BP) inferred using PROTGAMMALG model. All support values more than 80% BP and 0.8 PP or more than 50% BP and 0.5 PP are marked by full or open circles. Dashes ‘-’ indicate the support values < 50% BP or 0.5 PP. The branches and clades are assigned to the numbers that represent specified domain combinations listed in Figure 1. The alignment used to construct this phylogeny can be downloaded from http://www.mn.uio.no/bio/english/people/aca/kamran/data/Calpain_Metazoa_accession.dat. Click here for file
