# Supplementary material for: Massive expansion of the calpain gene family in unicellular eukaryotes
Source: BMC Evol Biol. 2012 Sep 29;12:193. doi: 10.1186/1471-2148-12-193 (PMC3563603; doi:10.1186/1471-2148-12-193)
Supplement: Additional file 2 — Figure S1. Eukaryotic calpain phylogeny inferred from the CysPc domain alignment (247 calpain sequences and 202 positions). The phylogeny is obtained from the consensus between two independent Bayesian inferences. For each node, support values are marked (numbers from left to right: Bayesian posterior probabilities (PP) inferred under LG /CAT models and maximum-likelihood bootstraps (% BP) inferred using PROTGAMMALG model) if all are more than 80% BP and 0.8 PP (filled circle) or more than 50% BP and 0.5 PP (open circle). Dashes ‘-’ show the support values are marked< 50% BP or 0.5 PP. For multiple CysPc domains found in the calpain sequence,1st, 2nd and 3rd indicate their orders from N-terminus to C-terminus of the calpain sequence. The domains marked by ‘*’ indicate their identities only have marginal significance of e-value in domain database research due to high sequence divergence. The branches and clades are assigned to the numbers that represent specified domain combinations shown in Figure 1. [file 1471-2148-12-193-S2.pdf]

Fig. S1. Eukaryotic calpain phylogeny inferred from the CysPc domain alignment (247 calpain sequences and 202 positions). The phylogeny is obtained from the consensus between two independent Bayesian inferences. For each node, support values (Bayesian posterior probabilities (PP) inferred under LG (left) /CAT (middle) models and maximum-likelihood bootstraps (% BP) inferred using PROTGAMMALG (right) model) are marked if all are more than 80% BP and 0.8 PP (filled circle) or more than 50% BP and 0.5 PP (open circle). Dashes '-' show the support values < 50% BP or 0.5 PP. For multiple CysPc domains found in the calpain sequence, [1st], [2nd] and [3rd] indicate their orders from N-terminus to C-terminus of the calpain sequence. The domains marked by '*' indicate their identities only have marginal significance of e-value in domain database research due to high sequence divergence. The branches and clades are assigned to the numbers that represent specified domain combinations shown in Fig. 1.

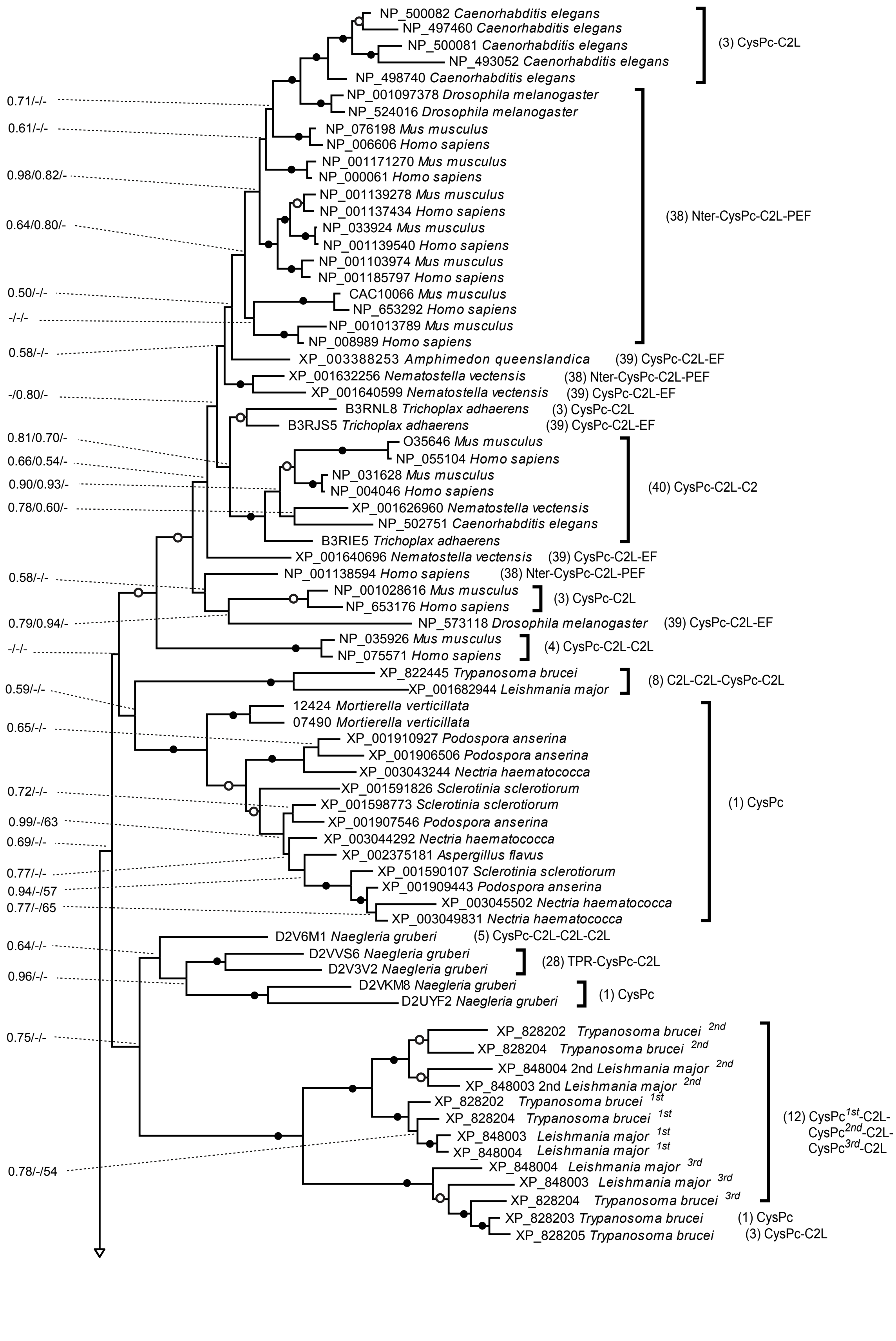

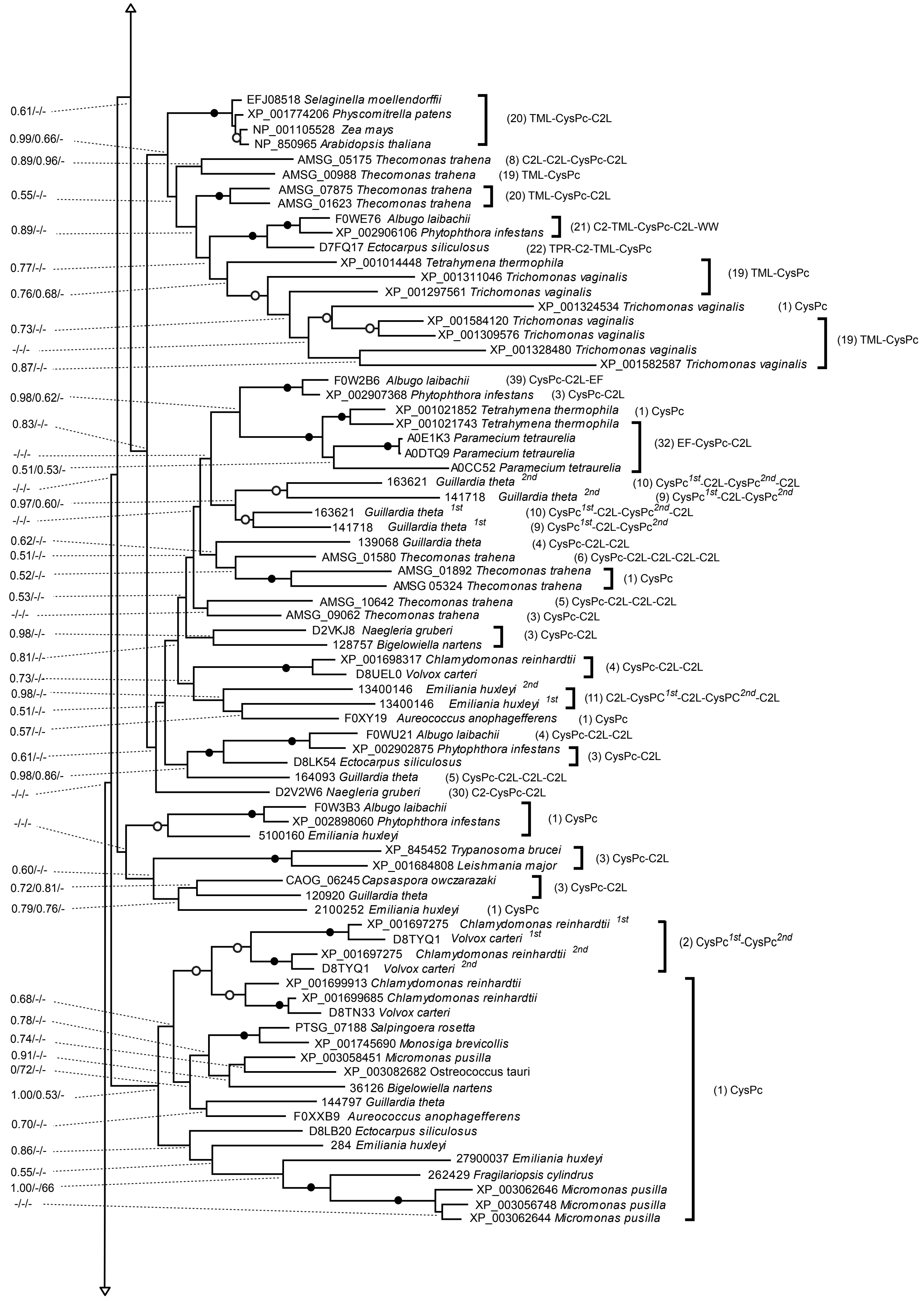

0.61/-/-
0.99/0.66/-
0.89/0.96/-
0.55/-/-
0.89/-/-
0.77/-/-
0.76/0.68/-
0.73/-/-
-/-/-
0.87/-/-
0.98/0.62/-
0.83/-/-
-/-/-
0.51/0.53/-
-/-/-
0.97/0.60/-
-/-/-
0.62/-/-
0.51/-/-
0.52/-/-
0.53/-/-
-/-/-
0.98/-/-
0.81/-/-
0.73/-/-
0.98/-/-
0.51/-/-
0.57/-/-
0.61/-/-
0.98/0.86/-
-/-/-
-/-/-
0.60/-/-
0.72/0.81/-
0.79/0.76/-
0.68/-/-
0.78/-/-
0.74/-/-
0.91/-/-
0/72/-/-
1.00/0.53/-
0.70/-/-
0.86/-/-
0.55/-/-
1.00/-/66
-/-/-
EFJ08518 Selaginella moellendorffii
XP_001774206 Physcomitrella patens
NP_001105528 Zea mays
NP_850965 Arabidopsis thaliana
(20) TML-CysPc-C2L
AMSG_05175 Thecomonas trahena (8) C2L-C2L-CysPc-C2L
AMSG_00988 Thecomonas trahena (19) TML-CysPc
AMSG_07875 Thecomonas trahena
AMSG_01623 Thecomonas trahena
(20) TML-CysPc-C2L
F0WE76 Albugo laibachii
XP_002906106 Phytophthora infestans
(21) C2-TML-CysPc-C2L-WW
D7FQ17 Ectocarpus siliculosus (22) TPR-C2-TML-CysPc
XP_001014448 Tetrahymena thermophila
XP_001311046 Trichomonas vaginalis
XP_001297561 Trichomonas vaginalis
(19) TML-CysPc
XP_001324534 Trichomonas vaginalis (1) CysPc
XP_001584120 Trichomonas vaginalis
XP_001309576 Trichomonas vaginalis
XP_001328480 Trichomonas vaginalis
XP_001582587 Trichomonas vaginalis
(19) TML-CysPc
F0W2B6 Albugo laibachii (39) CysPc-C2L-EF
XP_002907368 Phytophthora infestans (3) CysPc-C2L
XP_001021852 Tetrahymena thermophila (1) CysPc
XP_001021743 Tetrahymena thermophila
A0E1K3 Paramecium tetraurelia
A0DTQ9 Paramecium tetraurelia
A0CC52 Paramecium tetraurelia
(32) EF-CysPc-C2L
163621 Guillardia theta 2nd (10) CysPc1st-C2L-CysPc2nd-C2L
141718 Guillardia theta 2nd (9) CysPc1st-C2L-CysPc2nd
163621 Guillardia theta 1st (10) CysPc1st-C2L-CysPc2nd-C2L
141718 Guillardia theta 1st (9) CysPc1st-C2L-CysPc2nd
139068 Guillardia theta (4) CysPc-C2L-C2L
AMSG_01580 Thecomonas trahena (6) CysPc-C2L-C2L-C2L-C2L
AMSG_01892 Thecomonas trahena
AMSG 05324 Thecomonas trahena
(1) CysPc
AMSG_10642 Thecomonas trahena (5) CysPc-C2L-C2L-C2L
AMSG_09062 Thecomonas trahena (3) CysPc-C2L
D2VKJ8 Naegleria gruberi
128757 Bigelowiella nartens
(3) CysPc-C2L
XP_001698317 Chlamydomonas reinhardtii
D8UEL0 Volvox carteri
(4) CysPc-C2L-C2L
13400146 Emiliania huxleyi 2nd
13400146 Emiliania huxleyi 1st
(11) C2L-CysPC1st-C2L-CysPC2nd-C2L
F0XY19 Aureococcus anophagefferens (1) CysPc
F0WU21 Albugo laibachii (4) CysPc-C2L-C2L
XP_002902875 Phytophthora infestans
D8LK54 Ectocarpus siliculosus
(3) CysPc-C2L
164093 Guillardia theta (5) CysPc-C2L-C2L-C2L
D2V2W6 Naegleria gruberi (30) C2-CysPc-C2L
F0W3B3 Albugo laibachii
XP_002898060 Phytophthora infestans
5100160 Emiliania huxleyi
(1) CysPc
XP_845452 Trypanosoma brucei
XP_001684808 Leishmania major
(3) CysPc-C2L
CAOG_06245 Capsaspora owczarazaki
120920 Guillardia theta
(3) CysPc-C2L
2100252 Emiliania huxleyi (1) CysPc
XP_001697275 Chlamydomonas reinhardtii 1st
D8TYQ1 Volvox carteri 1st
XP_001697275 Chlamydomonas reinhardtii 2nd
D8TYQ1 Volvox carteri 2nd
(2) CysPc1st-CysPc2nd
XP_001699913 Chlamydomonas reinhardtii
XP_001699685 Chlamydomonas reinhardtii
D8TN33 Volvox carteri
PTSG_07188 Salpingoera rosetta
XP_001745690 Monosiga brevicollis
XP_003058451 Micromonas pusilla
XP_003082682 Ostreococcus tauri
36126 Bigelowiella nartens
144797 Guillardia theta
F0XXB9 Aureococcus anophagefferens
D8LB20 Ectocarpus siliculosus
284 Emiliania huxleyi
27900037 Emiliania huxleyi
262429 Fragilariopsis cylindrus
XP_003062646 Micromonas pusilla
XP_003056748 Micromonas pusilla
XP_003062644 Micromonas pusilla
(1) CysPc

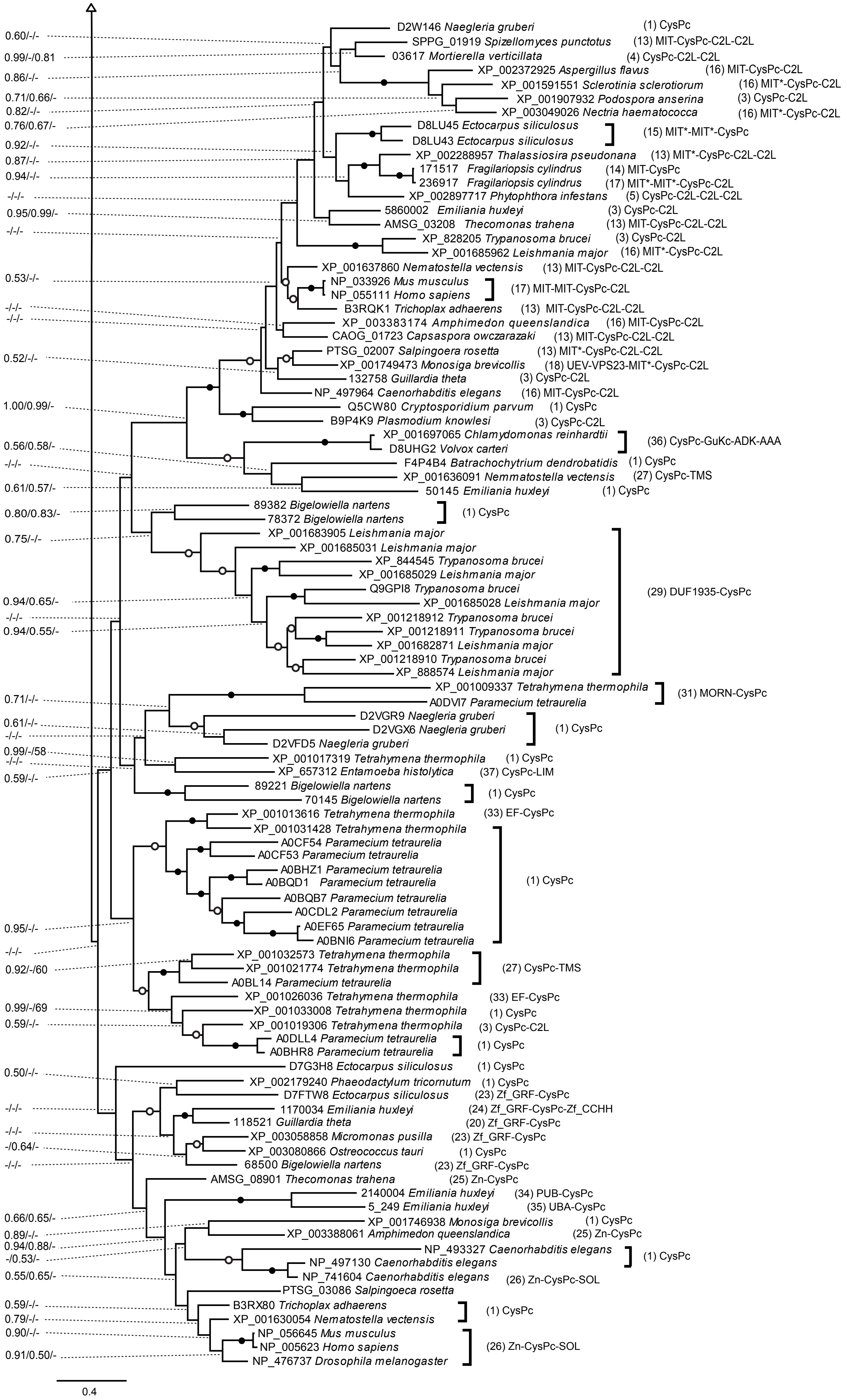

D2W146 Naegleria gruberi (1) CysPc
SPPG_01919 Spizellomyces punctotus (13) MIT-CysPc-C2L-C2L
03617 Mortierella verticillata (4) CysPc-C2L-C2L
XP_002372925 Aspergillus flavus (16) MIT-CysPc-C2L
XP_001591551 Sclerotinia sclerotiorum (16) MIT*-CysPc-C2L
XP_001907932 Podospora anserina (3) CysPc-C2L
XP_003049026 Nectria haematococca (16) MIT*-CysPc-C2L
D8LU45 Ectocarpus siliculosus
D8LU43 Ectocarpus siliculosus
(15) MIT*-MIT*-CysPc
XP_002288957 Thalassiosira pseudonana (13) MIT*-CysPc-C2L-C2L
171517 Fragilariopsis cylindrus (14) MIT-CysPc
236917 Fragilariopsis cylindrus (17) MIT*-MIT*-CysPc-C2L
XP_002897717 Phytophthora infestans (5) CysPc-C2L-C2L-C2L
5860002 Emiliania huxleyi (3) CysPc-C2L
AMSG_03208 Thecomonas trahena (13) MIT-CysPc-C2L-C2L
XP_828205 Trypanosoma brucei (3) CysPc-C2L
XP_001685962 Leishmania major (16) MIT*-CysPc-C2L
XP_001637860 Nematostella vectensis (13) MIT-CysPc-C2L-C2L
NP_033926 Mus musculus
NP_055111 Homo sapiens
(17) MIT-MIT-CysPc-C2L
B3RQK1 Trichoplax adhaerens (13) MIT-CysPc-C2L-C2L
XP_003383174 Amphimedon queenslandica (16) MIT-CysPc-C2L
CAOG_01723 Capsaspora owczarazaki (13) MIT-CysPc-C2L-C2L
PTSG_02007 Salpingoera rosetta (13) MIT*-CysPc-C2L-C2L
XP_001749473 Monosiga brevicollis (18) UEV-VPS23-MIT*-CysPc-C2L
132758 Guillardia theta (3) CysPc-C2L
NP_497964 Caenorhabditis elegans (16) MIT-CysPc-C2L
Q5CW80 Cryptosporidium parvum (1) CysPc
B9P4K9 Plasmodium knowlesi (3) CysPc-C2L
XP_001697065 Chlamydomonas reinhardtii
D8UHG2 Volvox carteri
(36) CysPc-GuKc-ADK-AAA
F4P4B4 Batrachochytrium dendrobatidis (1) CysPc
XP_001636091 Nemmatostella vectensis (27) CysPc-TMS
50145 Emiliania huxleyi (1) CysPc
89382 Bigelowiella nartens
78372 Bigelowiella nartens
(1) CysPc
XP_001683905 Leishmania major
XP_001685031 Leishmania major
XP_844545 Trypanosoma brucei
XP_001685029 Leishmania major
Q9GPI8 Trypanosoma brucei
XP_001685028 Leishmania major
XP_001218912 Trypanosoma brucei
XP_001218911 Trypanosoma brucei
XP_001682871 Leishmania major
XP_001218910 Trypanosoma brucei
XP_888574 Leishmania major
(29) DUF1935-CysPc
XP_001009337 Tetrahymena thermophila
A0DVI7 Paramecium tetraurelia
(31) MORN-CysPc
D2VGR9 Naegleria gruberi
D2VGX6 Naegleria gruberi
D2VFD5 Naegleria gruberi
(1) CysPc
XP_001017319 Tetrahymena thermophila (1) CysPc
XP_657312 Entamoeba histolytica (37) CysPc-LIM
89221 Bigelowiella nartens
70145 Bigelowiella nartens
(1) CysPc
XP_001013616 Tetrahymena thermophila (33) EF-CysPc
XP_001031428 Tetrahymena thermophila
A0CF54 Paramecium tetraurelia
A0CF53 Paramecium tetraurelia
A0BHZ1 Paramecium tetraurelia
A0BQD1 Paramecium tetraurelia
A0BQB7 Paramecium tetraurelia
A0CDL2 Paramecium tetraurelia
A0EF65 Paramecium tetraurelia
A0BNI6 Paramecium tetraurelia
(1) CysPc
XP_001032573 Tetrahymena thermophila
XP_001021774 Tetrahymena thermophila
A0BL14 Paramecium tetraurelia
(27) CysPc-TMS
XP_001026036 Tetrahymena thermophila (33) EF-CysPc
XP_001033008 Tetrahymena thermophila (1) CysPc
XP_001019306 Tetrahymena thermophila (3) CysPc-C2L
A0DLL4 Paramecium tetraurelia
A0BHR8 Paramecium tetraurelia
(1) CysPc
D7G3H8 Ectocarpus siliculosus (1) CysPc
XP_002179240 Phaeodactylum tricornutum (1) CysPc
D7FTW8 Ectocarpus siliculosus (23) Zf_GRF-CysPc
1170034 Emiliania huxleyi (24) Zf_GRF-CysPc-Zf_CCHH
118521 Guillardia theta (20) Zf_GRF-CysPc
XP_003058858 Micromonas pusilla (23) Zf_GRF-CysPc
XP_003080866 Ostreococcus tauri (1) CysPc
68500 Bigelowiella nartens (23) Zf_GRF-CysPc
AMSG_08901 Thecomonas trahena (25) Zn-CysPc
2140004 Emiliania huxleyi (34) PUB-CysPc
5_249 Emiliania huxleyi (35) UBA-CysPc
XP_001746938 Monosiga brevicollis (1) CysPc
XP_003388061 Amphimedon queenslandica (25) Zn-CysPc
NP_493327 Caenorhabditis elegans
NP_497130 Caenorhabditis elegans
(1) CysPc
NP_741604 Caenorhabditis elegans (26) Zn-CysPc-SOL
PTSG_03086 Salpingoeca rosetta
B3RX80 Trichoplax adhaerens
XP_001630054 Nematostella vectensis
(1) CysPc
NP_056645 Mus musculus
NP_005623 Homo sapiens
NP_476737 Drosophila melanogaster
(26) Zn-CysPc-SOL
0.60/-/-
0.99/-/0.81
0.86/-/-
0.71/0.66/-
0.82/-/-
0.76/0.67/-
0.92/-/-
0.87/-/-
0.94/-/-
-/-/-
0.95/0.99/-
-/-/-
0.53/-/-
-/-/-
-/-/-
0.52/-/-
1.00/0.99/-
0.56/0.58/-
-/-/-
0.61/0.57/-
0.80/0.83/-
0.75/-/-
0.94/0.65/-
-/-/-
0.94/0.55/-
0.71/-/-
0.61/-/-
-/-/-
0.99/-/58
-/-/-
0.59/-/-
0.95/-/-
-/-/-
0.92/-/60
0.99/-/69
0.59/-/-
0.50/-/-
-/-/-
-/-/-
-/0.64/-
-/-/-
0.66/0.65/-
0.89/-/-
0.94/0.88/-
-/0.53/-
0.55/0.65/-
0.59/-/-
0.79/-/-
0.90/-/-
0.91/0.50/-
0.4
