# Supplementary material for: Massive expansion of the calpain gene family in unicellular eukaryotes
Source: BMC Evol Biol. 2012 Sep 29;12:193. doi: 10.1186/1471-2148-12-193 (PMC3563603; doi:10.1186/1471-2148-12-193)
Supplement: Additional file 3 — Figure S2. Eukaryotic calpain phylogeny rooted by the bacterial outgroup (246 eukaryotic + 15 prokaryotic calpain sequences; 202 positions). The phylogeny is obtained from the consensus between two independent Bayesian inferences. For each node, support values are marked (numbers from left to right: Bayesian posterior probabilities (PP) inferred under LG (left) /CAT (middle) models and maximum-likelihood bootstraps (% BP) inferred using PROTGAMMALG (right) model) if all are more than 80% BP and 0.8 PP (filled circle) or more than 50% BP and 0.5 PP (open circle). Dashes ‘-’ show the support values < 50% BP or 0.5 PP. For multiple CysPc domains found in the calpain sequence,1st, 2nd and 3rd indicate their orders from N-terminus to C-terminus of the calpain sequence. The domains marked by ‘*’ indicate their identities only have marginal significance of e-value in domain database research due to high sequence divergence. The branches and clades are assigned to the numbers that represent specified domain combinations shown in Figure 1. The alignment used to build this phylogeny can be downloaded from http://www.mn.uio.no/bio/english/people/aca/kamran/data/Calpain_final_261_accession_outgroup.dat. [file 1471-2148-12-193-S3.pdf]

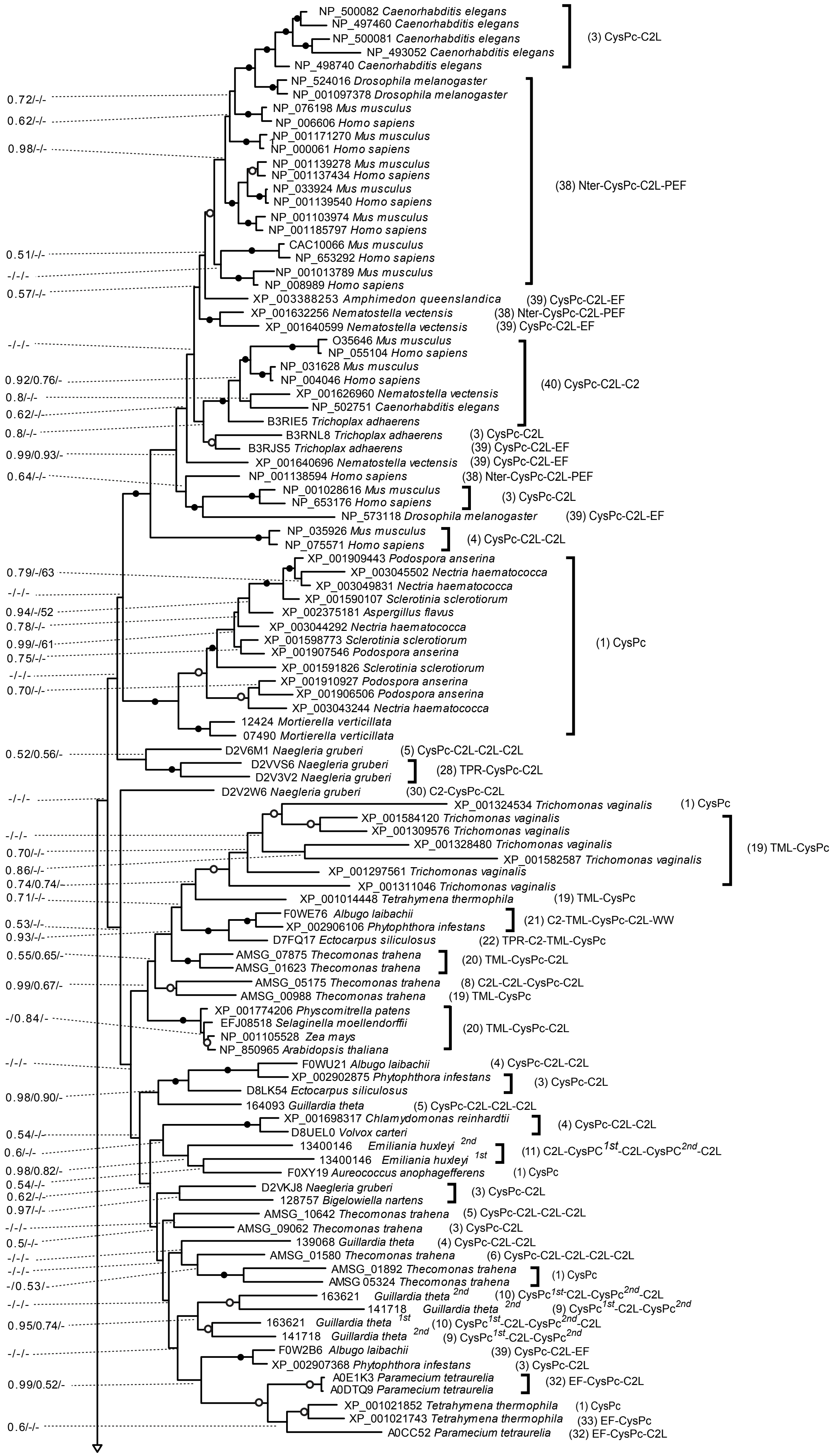

NP_500082 Caenorhabditis elegans
NP_497460 Caenorhabditis elegans
NP_500081 Caenorhabditis elegans
NP_493052 Caenorhabditis elegans
NP_498740 Caenorhabditis elegans
(3) CysPc-C2L
NP_524016 Drosophila melanogaster
NP_001097378 Drosophila melanogaster
NP_076198 Mus musculus
NP_006606 Homo sapiens
NP_001171270 Mus musculus
NP_000061 Homo sapiens
NP_001139278 Mus musculus
NP_001137434 Homo sapiens
NP_033924 Mus musculus
NP_001139540 Homo sapiens
NP_001103974 Mus musculus
NP_001185797 Homo sapiens
CAC10066 Mus musculus
NP_653292 Homo sapiens
NP_001013789 Mus musculus
NP_008989 Homo sapiens
(38) Nter-CysPc-C2L-PEF
XP_003388253 Amphimedon queenslandica (39) CysPc-C2L-EF
XP_001632256 Nematostella vectensis (38) Nter-CysPc-C2L-PEF
XP_001640599 Nematostella vectensis (39) CysPc-C2L-EF
O35646 Mus musculus
NP_055104 Homo sapiens
NP_031628 Mus musculus
NP_004046 Homo sapiens
XP_001626960 Nematostella vectensis
NP_502751 Caenorhabditis elegans
B3RIE5 Trichoplax adhaerens
(40) CysPc-C2L-C2
B3RNL8 Trichoplax adhaerens (3) CysPc-C2L
B3RJS5 Trichoplax adhaerens (39) CysPc-C2L-EF
XP_001640696 Nematostella vectensis (39) CysPc-C2L-EF
NP_001138594 Homo sapiens (38) Nter-CysPc-C2L-PEF
NP_001028616 Mus musculus
NP_653176 Homo sapiens
(3) CysPc-C2L
NP_573118 Drosophila melanogaster (39) CysPc-C2L-EF
NP_035926 Mus musculus
NP_075571 Homo sapiens
(4) CysPc-C2L-C2L
XP_001909443 Podospora anserina
XP_003045502 Nectria haematococca
XP_003049831 Nectria haematococca
XP_001590107 Sclerotinia sclerotiorum
XP_002375181 Aspergillus flavus
XP_003044292 Nectria haematococca
XP_001598773 Sclerotinia sclerotiorum
XP_001907546 Podospora anserina
XP_001591826 Sclerotinia sclerotiorum
XP_001910927 Podospora anserina
XP_001906506 Podospora anserina
XP_003043244 Nectria haematococca
12424 Mortierella verticillata
07490 Mortierella verticillata
(1) CysPc
D2V6M1 Naegleria gruberi (5) CysPc-C2L-C2L-C2L
D2VVS6 Naegleria gruberi
D2V3V2 Naegleria gruberi
(28) TPR-CysPc-C2L
D2V2W6 Naegleria gruberi (30) C2-CysPc-C2L
XP_001324534 Trichomonas vaginalis (1) CysPc
XP_001584120 Trichomonas vaginalis
XP_001309576 Trichomonas vaginalis
XP_001328480 Trichomonas vaginalis
XP_001582587 Trichomonas vaginalis
XP_001297561 Trichomonas vaginalis
XP_001311046 Trichomonas vaginalis
(19) TML-CysPc
XP_001014448 Tetrahymena thermophila (19) TML-CysPc
F0WE76 Albugo laibachii
XP_002906106 Phytophthora infestans
(21) C2-TML-CysPc-C2L-WW
D7FQ17 Ectocarpus siliculosus (22) TPR-C2-TML-CysPc
AMSG_07875 Thecomonas trahena
AMSG_01623 Thecomonas trahena
(20) TML-CysPc-C2L
AMSG_05175 Thecomonas trahena (8) C2L-C2L-CysPc-C2L
AMSG_00988 Thecomonas trahena (19) TML-CysPc
XP_001774206 Physcomitrella patens
EFJ08518 Selaginella moellendorffii
NP_001105528 Zea mays
NP_850965 Arabidopsis thaliana
(20) TML-CysPc-C2L
F0WU21 Albugo laibachii (4) CysPc-C2L-C2L
XP_002902875 Phytophthora infestans
D8LK54 Ectocarpus siliculosus
(3) CysPc-C2L
164093 Guillardia theta (5) CysPc-C2L-C2L-C2L
XP_001698317 Chlamydomonas reinhardtii
D8UEL0 Volvox carteri
(4) CysPc-C2L-C2L
13400146 Emiliania huxleyi ^{2nd}
13400146 Emiliania huxleyi ^{1st}
(11) C2L-CysPC^{1st}-C2L-CysPC^{2nd}-C2L
F0XY19 Aureococcus anophagefferens (1) CysPc
D2VKJ8 Naegleria gruberi
128757 Bigelowiella nartens
(3) CysPc-C2L
AMSG_10642 Thecomonas trahena (5) CysPc-C2L-C2L-C2L
AMSG_09062 Thecomonas trahena (3) CysPc-C2L
139068 Guillardia theta (4) CysPc-C2L-C2L
AMSG_01580 Thecomonas trahena (6) CysPc-C2L-C2L-C2L-C2L
AMSG_01892 Thecomonas trahena
AMSG 05324 Thecomonas trahena
(1) CysPc
163621 Guillardia theta ^{2nd} (10) CysPc^{1st}-C2L-CysPc^{2nd}-C2L
141718 Guillardia theta ^{2nd} (9) CysPc^{1st}-C2L-CysPc^{2nd}
163621 Guillardia theta ^{1st} (10) CysPc^{1st}-C2L-CysPc^{2nd}-C2L
141718 Guillardia theta ^{2nd} (9) CysPc^{1st}-C2L-CysPc^{2nd}
F0W2B6 Albugo laibachii (39) CysPc-C2L-EF
XP_002907368 Phytophthora infestans (3) CysPc-C2L
A0E1K3 Paramecium tetraurelia
A0DTQ9 Paramecium tetraurelia
(32) EF-CysPc-C2L
XP_001021852 Tetrahymena thermophila (1) CysPc
XP_001021743 Tetrahymena thermophila (33) EF-CysPc
A0CC52 Paramecium tetraurelia (32) EF-CysPc-C2L
0.72/-/-
0.62/-/-
0.98/-/-
0.51/-/-
-/-/-
0.57/-/-
-/-/-
0.92/0.76/-
0.8/-/-
0.62/-/-
0.8/-/-
0.99/0.93/-
0.64/-/-
0.79/-/63
-/-/-
0.94/-/52
0.78/-/-
0.99/-/61
0.75/-/-
-/-/-
0.70/-/-
0.52/0.56/-
-/-/-
-/-/-
0.70/-/-
0.86/-/-
0.74/0.74/-
0.71/-/-
0.53/-/-
0.93/-/-
0.55/0.65/-
0.99/0.67/-
-/0.84/-
-/-/-
0.98/0.90/-
0.54/-/-
0.6/-/-
0.98/0.82/-
0.54/-/-
0.62/-/-
0.97/-/-
-/-/-
0.5/-/-
-/-/-
-/-/-
-/0.53/-
-/-/-
0.95/0.74/-
-/-/-
0.99/0.52/-
0.6/-/-

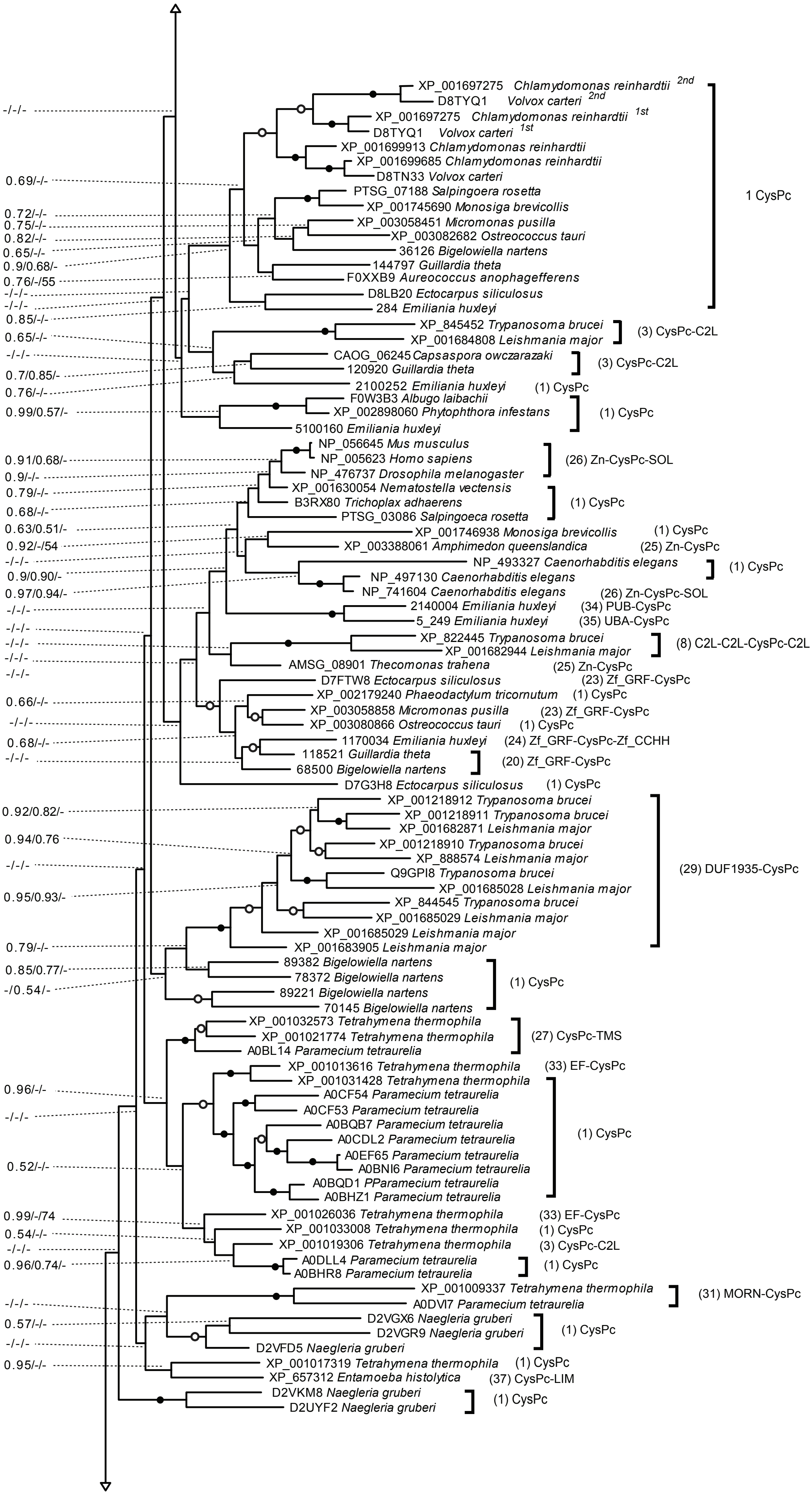
XP_001697275 Chlamydomonas reinhardtii 2nd
D8TYQ1 Volvox carteri 2nd
XP_001697275 Chlamydomonas reinhardtii 1st
D8TYQ1 Volvox carteri 1st
XP_001699913 Chlamydomonas reinhardtii
XP_001699685 Chlamydomonas reinhardtii
D8TN33 Volvox carteri
PTSG_07188 Salpingoera rosetta
XP_001745690 Monosiga brevicollis
XP_003058451 Micromonas pusilla
XP_003082682 Ostreococcus tauri
36126 Bigelowiella nartens
144797 Guillardia theta
F0XXB9 Aureococcus anophagefferens
D8LB20 Ectocarpus siliculosus
284 Emiliania huxleyi
1 CysPc
XP_845452 Trypanosoma brucei
XP_001684808 Leishmania major
(3) CysPc-C2L
CAOG_06245 Capsaspora owczarazaki
120920 Guillardia theta
(3) CysPc-C2L
2100252 Emiliania huxleyi
(1) CysPc
F0W3B3 Albugo laibachii
XP_002898060 Phytophthora infestans
5100160 Emiliania huxleyi
(1) CysPc
NP_056645 Mus musculus
NP_005623 Homo sapiens
NP_476737 Drosophila melanogaster
(26) Zn-CysPc-SOL
XP_001630054 Nematostella vectensis
B3RX80 Trichoplax adhaerens
PTSG_03086 Salpingoeca rosetta
(1) CysPc
XP_001746938 Monosiga brevicollis
(1) CysPc
XP_003388061 Amphimedon queenslandica
(25) Zn-CysPc
NP_493327 Caenorhabditis elegans
(1) CysPc
NP_497130 Caenorhabditis elegans
NP_741604 Caenorhabditis elegans
(26) Zn-CysPc-SOL
2140004 Emiliania huxleyi
(34) PUB-CysPc
5_249 Emiliania huxleyi
(35) UBA-CysPc
XP_822445 Trypanosoma brucei
XP_001682944 Leishmania major
(8) C2L-C2L-CysPc-C2L
AMSG_08901 Thecomonas trahena
(25) Zn-CysPc
D7FTW8 Ectocarpus siliculosus
(23) Zf_GRF-CysPc
XP_002179240 Phaeodactylum tricornutum
(1) CysPc
XP_003058858 Micromonas pusilla
(23) Zf_GRF-CysPc
XP_003080866 Ostreococcus tauri
(1) CysPc
1170034 Emiliania huxleyi
(24) Zf_GRF-CysPc-Zf_CCHH
118521 Guillardia theta
68500 Bigelowiella nartens
(20) Zf_GRF-CysPc
D7G3H8 Ectocarpus siliculosus
(1) CysPc
XP_001218912 Trypanosoma brucei
XP_001218911 Trypanosoma brucei
XP_001682871 Leishmania major
XP_001218910 Trypanosoma brucei
XP_888574 Leishmania major
Q9GPI8 Trypanosoma brucei
XP_001685028 Leishmania major
XP_844545 Trypanosoma brucei
XP_001685029 Leishmania major
XP_001685029 Leishmania major
XP_001683905 Leishmania major
(29) DUF1935-CysPc
89382 Bigelowiella nartens
78372 Bigelowiella nartens
89221 Bigelowiella nartens
70145 Bigelowiella nartens
(1) CysPc
XP_001032573 Tetrahymena thermophila
XP_001021774 Tetrahymena thermophila
A0BL14 Paramecium tetraurelia
(27) CysPc-TMS
XP_001013616 Tetrahymena thermophila
(33) EF-CysPc
XP_001031428 Tetrahymena thermophila
A0CF54 Paramecium tetraurelia
A0CF53 Paramecium tetraurelia
A0BQB7 Paramecium tetraurelia
A0CDL2 Paramecium tetraurelia
A0EF65 Paramecium tetraurelia
A0BNI6 Paramecium tetraurelia
A0BQD1 PParamecium tetraurelia
A0BHZ1 Paramecium tetraurelia
(1) CysPc
XP_001026036 Tetrahymena thermophila
(33) EF-CysPc
XP_001033008 Tetrahymena thermophila
(1) CysPc
XP_001019306 Tetrahymena thermophila
(3) CysPc-C2L
A0DLL4 Paramecium tetraurelia
A0BHR8 Paramecium tetraurelia
(1) CysPc
XP_001009337 Tetrahymena thermophila
A0DVI7 Paramecium tetraurelia
(31) MORN-CysPc
D2VGX6 Naegleria gruberi
D2VGR9 Naegleria gruberi
D2VFD5 Naegleria gruberi
(1) CysPc
XP_001017319 Tetrahymena thermophila
(1) CysPc
XP_657312 Entamoeba histolytica
(37) CysPc-LIM
D2VKM8 Naegleria gruberi
D2UYF2 Naegleria gruberi
(1) CysPc
-/-/-
0.69/-/-
0.72/-/-
0.75/-/-
0.82/-/-
0.65/-/-
0.9/0.68/-
0.76/-/55
-/-/-
-/-/-
0.85/-/-
0.65/-/-
-/-/-
0.7/0.85/-
0.76/-/-
0.99/0.57/-
0.91/0.68/-
0.9/-/-
0.79/-/-
0.68/-/-
0.63/0.51/-
0.92/-/54
-/-/-
0.9/0.90/-
0.97/0.94/-
-/-/-
-/-/-
-/-/-
-/-/-
-/-/-
0.66/-/-
-/-/-
0.68/-/-
-/-/-
0.92/0.82/-
0.94/0.76
-/-/-
0.95/0.93/-
0.79/-/-
0.85/0.77/-
-/0.54/-
0.96/-/-
-/-/-
0.52/-/-
0.99/-/74
0.54/-/-
-/-/-
0.96/0.74/-
-/-/-
0.57/-/-
-/-/-
0.95/-/-

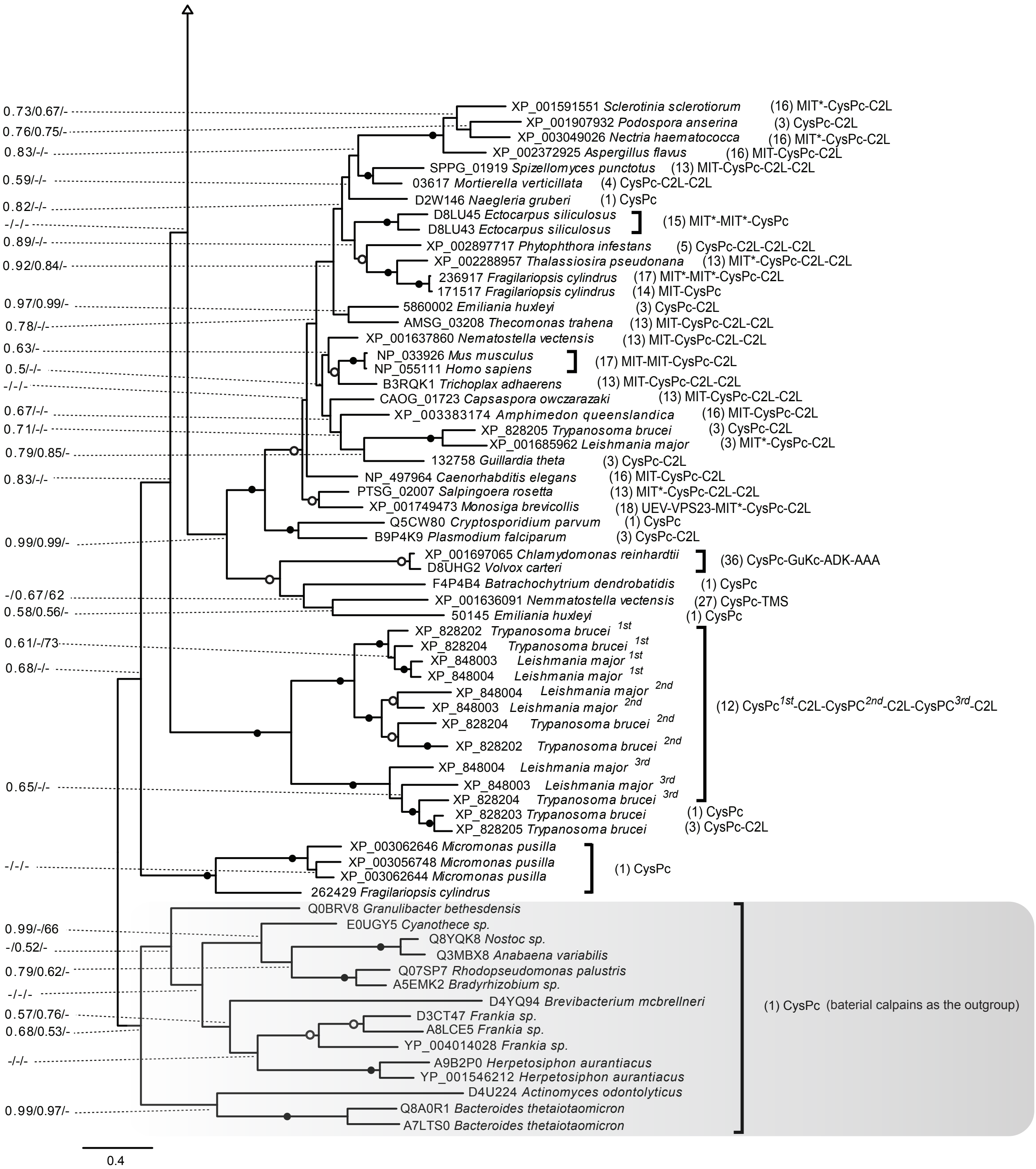

Fig. S2. Eukaryotic calpain phylogeny rooted by the bacterial outgroup (246 eukaryotic + 15 prokaryotic calpain sequences; 202 positions). The phylogeny is obtained from the consensus between two independent Bayesian inferences. For each node, support values (Bayesian posterior probabilities (PP) inferred under LG (left) /CAT (middle) models and maximum-likelihood bootstraps (% BP) inferred using PROTGAMMALG (right) model) are marked if all are more than 80% BP and 0.8 PP (filled circle) or more than 50% BP and 0.5 PP (open circle). Dashes '-' show the support values < 50% BP or 0.5 PP. For multiple CysPc domains found in the calpain sequence, 1st, 2nd and 3rd indicate their orders from N-terminus to C-terminus of the calpain sequence. The domains marked by '*' indicate their identities only have marginal significance of e-value in domain database research due to high sequence divergence. The branches and clades are assigned to the numbers that represent specified domain combinations shown in Fig. 1. The alignment used to build this phylogeny can be downloaded from http://folk.uio.no/senz/Calpain_final_261_accession_outgroup.dat
