# Supplementary material for: Massive expansion of the calpain gene family in unicellular eukaryotes
Source: BMC Evol Biol. 2012 Sep 29;12:193. doi: 10.1186/1471-2148-12-193 (PMC3563603; doi:10.1186/1471-2148-12-193)
Supplement: Additional file 5 — Figure S4. Proposed origin of calpain superfamily domain combinations shown on the global eukaryote phylogeny. The tree is rooted according to Cavalier-Smith T 2010. Biol Lett 6(3):342-345. Red bars on the branches indicate the hypothetical origin of specified domain combinations. Calpain variants only found in one taxon or among closely related species are marked in red within the supergroup rectangles, which likely constitute lineage-specific domain combinations. [file 1471-2148-12-193-S5.pdf]

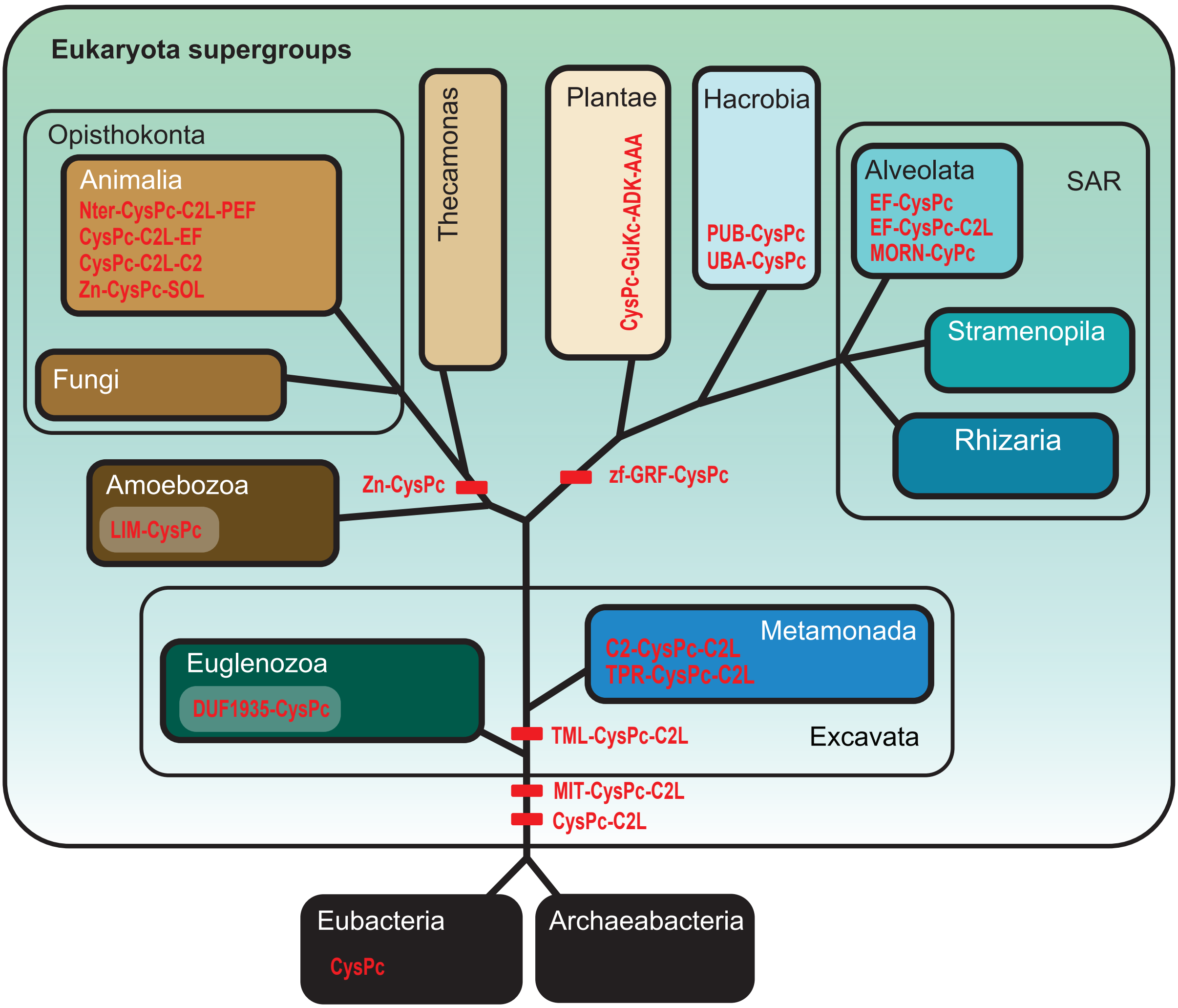

Fig. S4. Proposed origin of calpain superfamily domain combinations shown on the global eukaryote phylogeny (Cavalier-Smith T 2010. Biol Lett 6(3):342-345). Red bars on the braches indicate the hypothetical origin of specified domain combinations. Calpain variants only found in one taxon or among closely related species are marked in red within the supergroup rectangles, which likely constitute lineage-specific domain combinations.
