# Supplementary material for: Massive expansion of the calpain gene family in unicellular eukaryotes
Source: BMC Evol Biol. 2012 Sep 29;12:193. doi: 10.1186/1471-2148-12-193 (PMC3563603; doi:10.1186/1471-2148-12-193)

Fig. S3. Phylogeny of the calpain genes with MIT modules. The tree is obtained from the consensus between two independent Bayesian inferences. Support values at the nodes represent Bayesian posterior probabilities (PP) inferred under CAT (left) /LG (middle) models and the maximum-likelihood bootstraps (% BP) inferred using PROTGAMMALG (right) model in RAxML v7.2.5. All support values more than 80% BP and 0.8 PP or more than 50% BP and 0.5 PP are shown as full or open circles. Dashes '-' show the support values < 50% or 0.5 PP. The MIT calpain genes found in *Tetraodon nigroviridis, Takifugu rubripes, Gasterosteus aculeatus, Gadus morhua, Salmo salar, Danio rerio, Oryzia latipes, Gallus gallus, Dasypus novemcinctus, Ailuropoda melanoleuca, Sus scrofa, Canis familiaris, Bos taurus and Rattus norvegicus* are added to this analysis. The domains marked by '*' indicate their identities only have marginal significance of e-value in domain database research due to high sequence divergence. The branches and clades are assigned to the numbers that correspond to specified domain combinations shown in Fig. 1. The alignment used to reconstruct the phylogeny of MIT calpains can be downloaded from http://folk.uio.no/senz/Calpain_MIT_accession.dat.

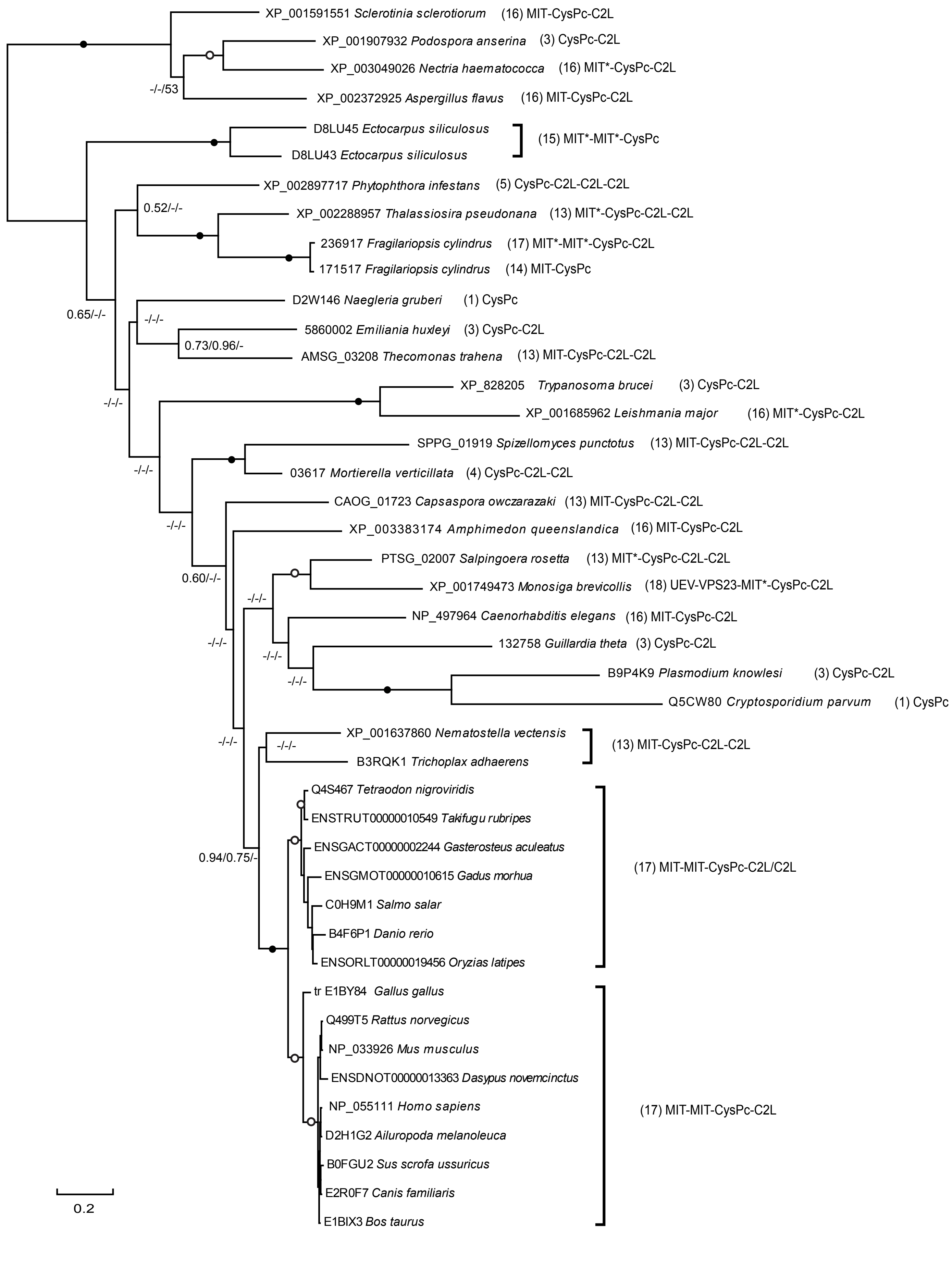

Supplement: Additional file 4 — Figure S3. Phylogeny of the calpain genes with MIT modules. The tree is obtained from the consensus between two independent Bayesian inferences. Support values are marked at the nodes, and numbers from left to right represent Bayesian posterior probabilities (PP) inferred under CAT /LG models and the maximum-likelihood bootstraps (% BP) inferred using PROTGAMMALG model in RAxML v7.2.5. All support values more than 80% BP and 0.8 PP or more than 50% BP and 0.5 PP are shown as full or open circles. Dashes ‘-’ show the support values < 50% or 0.5 PP. The MIT calpain genes found in Tetraodon nigroviridis, Takifugu rubripes, Gasterosteus aculeatus, Gadus morhua, Salmo salar, Danio rerio, Oryzia latipes, Gallus gallus, Dasypus novemcinctus, Ailuropoda melanoleuca, Sus scrofa, Canis familiaris, Bos taurus and Rattus norvegicus are added to this analysis. The domains marked by ‘*’ indicate their identities only have marginal significance of e-value in domain database research due to high sequence divergence. The branches and clades are assigned to the numbers that correspond to specified domain combinations shown in Figure 1. The alignment used to reconstruct the phylogeny of MIT calpains can be downloaded from http://www.mn.uio.no/bio/english/people/aca/kamran/data/Calpain_MIT_accession.dat. [file 1471-2148-12-193-S4.pdf]
