# Supplementary material for: Massive expansion of the calpain gene family in unicellular eukaryotes
Source: BMC Evol Biol. 2012 Sep 29;12:193. doi: 10.1186/1471-2148-12-193 (PMC3563603; doi:10.1186/1471-2148-12-193)

Fig. S5. Phylogeny of calpains in Metazoa. The topology is obtained from the consensus between two independent Bayesian inferences. The representative taxa sampled here include *Homo species*, *Mus musculus*, *Gallus gallus*, *Xenopus laevis*, *Danio rerio*, *Nematostella vectensis*, *Drosophila melanogaster, Caenorhabditis elegans, Trichoplas adhaerens* and *Amphimedon queenslandica*. Support values at the nodes show Bayesian posterior probabilities (PP) inferred under CAT (left) /LG (middle) models and the maximum-likelihood bootstraps (% BP) inferred using PROTGAMMALG (right) model. All support values more than 80% BP and 0.8 PP or more than 50% and 0.5 PP are marked by full or open circles. Dashes '-' indicate the support values < 50% or 0.5 PP. The branches and clades are assigned to the numbers that represent specified domain combinations listed in Fig. 1. The alignment used to construct this phylogeny can be downloaded from http://folk.uio.no/senz/Calpain_Metazoa_accession.dat.

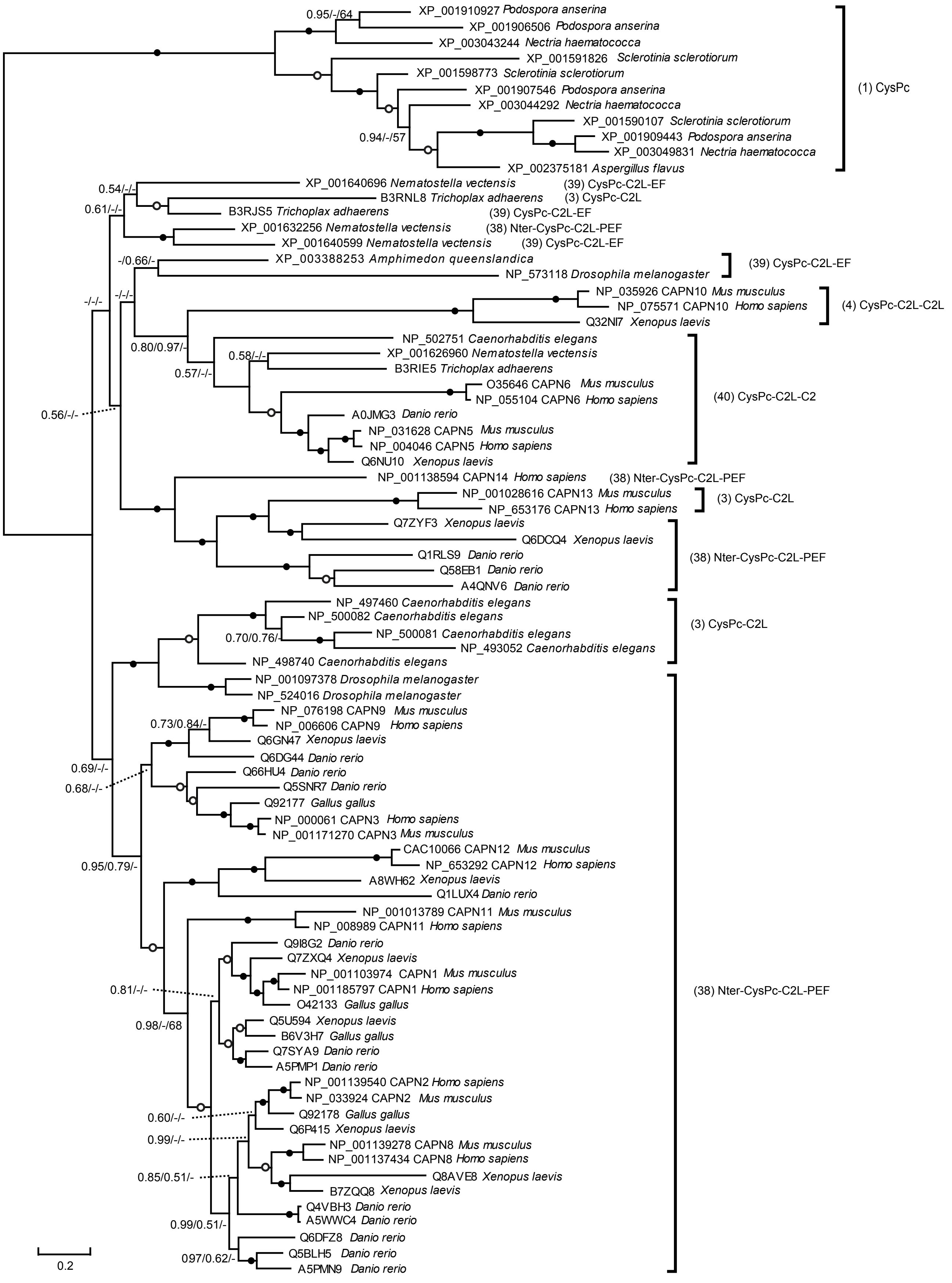

Supplement: Additional file 6 — Figure S5. Phylogeny of calpains in Metazoa. The topology is obtained from the consensus between two independent Bayesian inferences. The representative taxa sampled here include Homo species, Mus musculus, Gallus gallus, Xenopus laevis, Danio rerio, Nematostella vectensis, Drosophila melanogaster, Caenorhabditis elegans, Trichoplas adhaerens and Amphimedon queenslandica. Support values are marked at the nodes, and number from left to right represent Bayesian posterior probabilities (PP) inferred under CAT /LG models and the maximum-likelihood bootstraps (% BP) inferred using PROTGAMMALG model. All support values more than 80% BP and 0.8 PP or more than 50% BP and 0.5 PP are marked by full or open circles. Dashes ‘-’ indicate the support values < 50% BP or 0.5 PP. The branches and clades are assigned to the numbers that represent specified domain combinations listed in Figure 1. The alignment used to construct this phylogeny can be downloaded from http://www.mn.uio.no/bio/english/people/aca/kamran/data/Calpain_Metazoa_accession.dat. [file 1471-2148-12-193-S6.pdf]
